# Furosemide and Serum Protein-Bound Uremic Toxin Concentrations in Patients With CKD

**DOI:** 10.1016/j.ekir.2025.04.040

**Published:** 2025-05-02

**Authors:** Margaux Costes-Albrespic, Natalia Alencar de Pinho, Islam Amine Larabi, Carolla El Chamieh, Solène M. Laville, Denis Fouque, Maurice Laville, Luc Frimat, Jean-Claude Alvarez, Ziad A. Massy, Sophie Liabeuf, CKD-REIN Study Group, Natalia Alencar De Pinho, Dorothée Cannet, Christian Combe, Denis Fouque, Luc Frimat, Aghilès Hamroun, Yves-Edouard Herpe, Christian Jacquelinet, Maurice Laville, Sophie Liabeuf, Ziad A. Massy, Pascal Morel, Christophe Pascal, Roberto Pecoits-Filho, Joost Schanstra, Bénédicte Stengel, Céline Lange, Oriane Lambert, Marie Metzger

**Affiliations:** 1 Clinical Epidemiology Team, Centre for Research in Epidemiology and Population Health (CESP), Inserm U1018, Paris-Saclay University, Versailles Saint-Quentin University, Villejuif, France; 2 Pharmaco-epidemiology Unit, Department of Clinical Pharmacology, Amiens-Picardie University Medical Center, Amiens, France; 3 MP3CV Laboratory, Jules Verne University of Picardie, Amiens, France; 4 Nephrology Department, Lyon-Sud University Hospital, Claude Bernard Lyon 1 University, Pierre-Bénite, France; 5 CarMeN laboratory, INSERM U1060, INRAE U1397, Claude Bernard University Lyon 1, Pierre-Bénite, France; 6 Nephrology Department, University Regional Hospital of Nancy, Vandoeuvre-lès-Nancy, France; 7 APEMAC, University of Lorraine, Nancy, France; 8 AURA (Association pour l'Utilisation du Rein Artificiel), Paris, France; 9 Nephrology Department, Ambroise Paré University Hospital, APHP, Boulogne-Billancourt, Paris, France; 10 Department of Pharmacology and Toxicology, CARNOT Personalized medicine platform, Raymond Poincaré Hospital, AP-HP, Garches, France; 11 Équipe MOODS, CESP, Inserm U1018, Université Paris-Saclay, Versailles Saint-Quentin University, Garches, France

**Keywords:** CKD, furosemide, loop diuretic, OAT inhibitor, organic anion transporter, uremic toxin

## Abstract

**Introduction:**

Furosemide is commonly prescribed to patients with chronic kidney disease (CKD) but may impair the kidney’s excretion of protein-bound uremic toxins (PBUTs) via the organic anion transporters 1 and 3 (OAT1 and OAT3). We evaluated the association between furosemide prescription (status and dose level) and the serum concentrations of free OAT1/3-inhibiting uremic toxins (UTs) in patients with CKD.

**Methods:**

We included 2342 patients with CKD (stages 2–5) from the CKD–Renal Epidemiology and Information Network (CKD-REIN) cohort and with centralized serum UT assay data at baseline. The UTs were assayed using liquid chromatography - tandem mass spectrometry. The OAT1/3-inhibiting UTs identified in a literature review included indoxyl sulphate (IS), kynurenine (Kyn), p-cresyl sulphate (PCS), and indole-3-acetic acid (IAA). Multiple linear regression was used to assess each PBUT or their sum (ΣUTs_free_) as the dependent variable.

**Results:**

Patients prescribed furosemide (*n* = 799, 34%) were older and had a lower estimated glomerular filtration rate (eGFR), a higher C-reactive protein (CRP) concentration, more comorbidities, and more concomitant medications than patients not prescribed furosemide. After adjustment for potential confounders, patients prescribed > 120 mg furosemide had significantly higher serum concentrations of ΣUTs_free_ (+19.1%), IS (+31.9%), Kyn (+9.3%), PCS (+29.3%), and IAA (+162.9%) than patients not prescribed furosemide. Using a smooth function to model the association between the furosemide dose level and PBUTs, we observed (for ΣUTs_free_ and each free UT) a steep increase between 80 and 100 mg and then a high plateau.

**Conclusion:**

In patients with CKD, furosemide (particularly at a dose level > 120 mg) is independently associated with higher serum free PBUT concentrations. Our findings suggest that drug-UT competition contributes to PBUT accumulation.

## Graphical abstract

**Figure:**
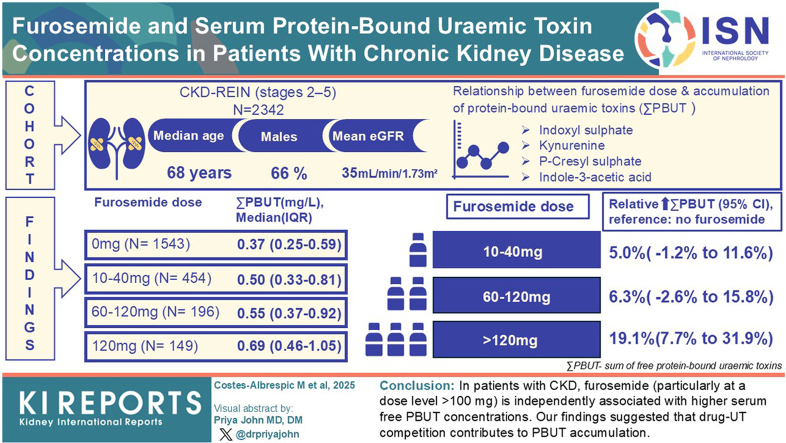

See Commentary on Page 2094

CKD is an increasingly important public health issue, with an estimated worldwide prevalence of 11% to 13%.^1^ The prevention and management of CKD progression are major challenges because the latter increases morbidity and mortality rates and is a major risk factor for cardiovascular (CV) disease.^2^

As kidney function declines in patients with CKD, several solutes that are usually excreted by healthy kidneys are retained in the body. When an accumulated solute impairs one or more biological functions, it is referred to as a UT.^3^, ^4^, ^5^ UTs are categorized as (i) small water-soluble molecules, (ii) protein-bound compounds, or (iii) middle molecules. UTs are nontraditional CKD-related risk factors and contribute to CKD progression, CV morbidity, CV mortality, and bone dysfunction.^6^^,^^7^ Moreover, UTs are often considered to be the main cause of the CKD-associated symptom burden.^8^^,^^9^ It is therefore essential to identify factors that influence UT concentrations.

Given their ability to bind to albumin, PBUTs are predominantly eliminated through specific influx channels in the proximal renal tubules, such as the OAT1 and/or OAT3.^10^ The results of *in vivo* and *in vitro* studies have shown that IS, Kyn, PCS, and IAA are excreted by both OAT1 and OAT3, whereas kynurenate (KA) is excreted by OAT1 only.^11^, ^12^, ^13^, ^14^, ^15^, ^16^, ^17^, ^18^, ^19^, ^20^

CKD is associated with a higher risk of premature mortality and CV disease and a higher prevalence of coexisting diseases. To manage all these complications, patients with CKD often have to take 52 or more medications concomitantly.^21^ This polypharmacy results in a greater frequency of adverse drug reactions and can increase the likelihood of pharmacokinetic interactions between drugs and UTs.^22^ Many PBUTs and drugs prescribed to patients with CKD are transported from the blood into the urinary tract through the OAT system.^23^^,^^24^ The OATs are mainly expressed in the liver, brain, and kidney and are known to be involved in the transport of proton-pump inhibitors, diuretics, nonsteroidal antiinflammatory drugs, antiviral drugs, and antibiotics.^23^^,^^25^^,^^26^ A few studies have shown that inhibition of OAT1 or OAT3 by a drug induces the accumulation of PBUTs.^24^^,^^27^, ^28^, ^29^, ^30^

The loop diuretic, furosemide is widely prescribed for managing blood pressure and hypervolemia in patients with CKD. Importantly, OAT1 and OAT3 are involved in the transport of furosemide. These 2 transporters have similar affinities and inhibition potencies for furosemide, and the results of an *in vivo* study in the mouse indicated that OAT1 and OAT3 contribute equally to furosemide excretion.^31^, ^32^, ^33^, ^34^

Therefore, we hypothesized that (i) dose-dependent competition between furosemide and selected PBUTs (IS, Kyn, PCS, IAA, and KA) for OAT1 and/or OAT3 leads to higher serum free UT concentrations in furosemide-treated patients with CKD than in those not treated with furosemide, and (ii) this difference is independent of other determinants of UT concentrations, such as the eGFR (Figure 1). To the best of our knowledge, the association between furosemide prescription and serum PBUT concentrations has not previously been investigated in nondialyzed patients with CKD. The objective of the present study was to investigate the association between furosemide prescription and serum PBUT concentrations in patients with CKD participating in the CKD-REIN study.^35^

**Figure 1:**
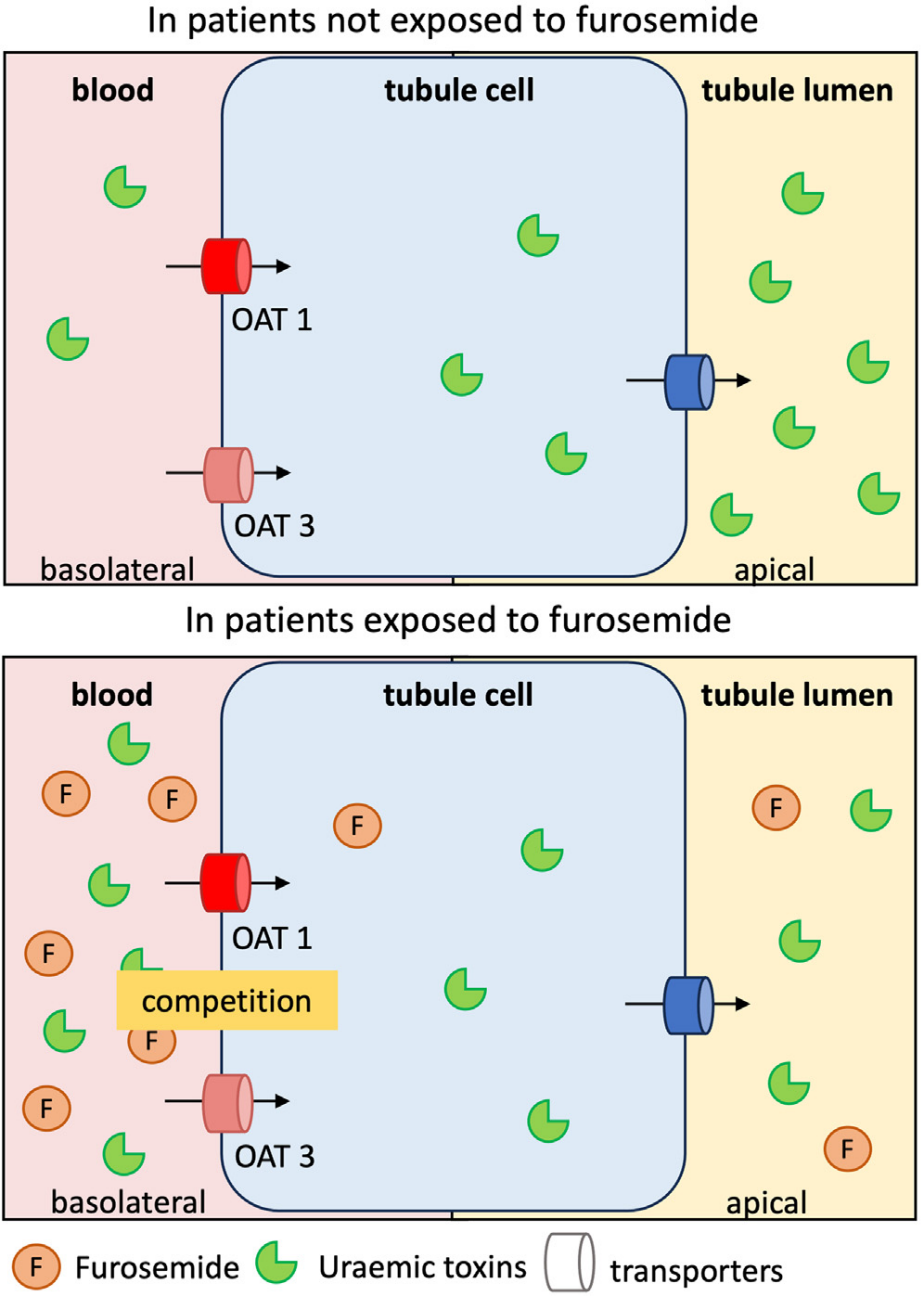
A schematic diagram of the putative competition between furosemide and protein-bound uremic toxins in chronic kidney disease. In patients with a furosemide prescription, competition for OAT1 and OAT3 might lead to reduced tubular excretion of PBUTs and thus elevated serum PBUT concentrations. OAT, organic anion transporter; PBUT, protein-bound uremic toxin.

## Methods

### Data Source and Population

The CKD-REIN is a prospective cohort study conducted in France. The study is nationally representative in terms of the geographical location and legal status of the participating centers. From 2013 to 2016, we included 3033 patients with moderate-to-advanced CKD and who were not on maintenance dialysis and had not been transplanted. The CKD-REIN study’s rationale, design, and methods have been described in detail elsewhere.^18^ The protocol was approved by the French National Institute of Health and Medical Research's independent ethics committee (CEE IRB00003888 (Paris, France) on June 13, 2013, and the study was registered at ClinicalTrials.gov (NCT03381950). All the study participants were aged ≥18 and provided their written, informed consent.

In the present analysis, we included patients for whom a serum sample had been collected within 3 months of inclusion and who had UT assay data (*n* = 2406). Patients with missing data on the prescription of potential OAT1/OAT3–inhibiting drugs and/or the prescribed dose level of furosemide were excluded (*n* = 64). Therefore, a total of 2342 patients were included in the final analysis (Supplementary Figure S1).

### Assessment of Furosemide Prescriptions

Participants were asked to bring all their drug prescriptions (issued by any physician) from the past 3 months to the inclusion visit. The drugs were then coded by clinical research associates using an electronic case report form linked to the international Anatomical Therapeutic and Chemical thesaurus.^36^ For each drug prescription, the trade name, international nonproprietary name, Anatomical Therapeutic and Chemical class, unit dose, prescribed daily dose level, pharmaceutical formulation, and administration route were available. We chose furosemide (Anatomical Therapeutic and Chemical C03CA01) as a drug known to inhibit OAT1 and OAT3 at therapeutic concentrations and that is commonly prescribed to patients with CKD. Furosemide strongly inhibits OAT1 and OAT3.^31^, ^32^, ^33^, ^34^ Patients who received other loop diuretics than furosemide (bumetanide [*n* = 25] and piretanide [*n* = 1]) were classified into the “no prescription” group.

### Covariates

Baseline data (including sociodemographic characteristics, medical histories, and laboratory data) were collected from interviews, medical records, and self-questionnaires by clinical research associates. Sex was defined based on assignment at birth (men or women). Diabetes was defined as the prescription of a glucose-lowering drug, a glycated hemoglobin concentration ≥ 6.5%, a fasting glucose concentration ≥ 7 mmol/, or a nonfasting glucose concentration ≥ 11 mmol/l. The CV history at baseline was assessed through medical records and included heart failure, coronary artery disease, cerebrovascular disease, peripheral arterial disease, and cardiac rhythm disorders. Any history of acute kidney injury was recorded. Routine laboratory data were recorded in hospital central laboratories and/or private medical laboratories as part of the patients’ usual care. The urine albumin-to-creatinine ratio was measured or was estimated from the protein-to-creatinine ratio.^37^ Height and weight data recorded by nephrologists or outpatient nurses during a routine visit were used to calculate the body mass index (kg/m^2^). Prescriptions of potential OAT1 or 3 inhibitors included thiazides, thiazide-like diuretics, proton-pump inhibitors, and angiotensin II receptor blockers.

### Serum Concentrations of UTs, and Other Centralized Measurements

At baseline, serum samples were collected from fasting patients in the morning, immediately stored at 4 °C, and aliquoted within 6 hours without additional processing. The samples were stored at −80 °C in a biological resource center (Biobanque de Picardie, BRIF number: BB-0033-00017, Amiens, France) and shipped frozen to Paris (France) for analysis. The staff at each laboratory were blinded to the outcomes and the patients’ characteristics. Serum concentrations of CRP and albumin were measured centrally. Serum CRP concentrations were assayed on a chemistry analyzer (Architect C16000, Abbott, Chicago, IL), and serum albumin concentrations were measured by immunoturbidimetry (Atellica CH, Siemens, Erlangen, Germany). eGFR was estimated using the 2009 CKD Epidemiology Collaboration equation. The centralized isotope dilution mass spectrometry-traceable creatinine concentration was determined with enzyme assays. UT fractions in serum were assayed using a validated ultra-high-performance liquid chromatography tandem mass spectrometry technique, as described previously.^38^ To determine total UT concentrations, a 50-μl serum sample was precipitated with 340 μl of methanol plus 25 μl of isotope-labelled internal standards. After centrifugation for 10 minutes at 9000 g, the supernatant was evaporated under a nitrogen stream and then reconstituted in 80 μl of water. Free UT concentrations were determined by ultrafiltration; 150 μl of serum was introduced into an ultracentrifugal filter (pore cut-off: 30 kDa) and centrifuged at 13,300 g for 20 minutes. Given that PBUTs are mainly bound to albumin (which weighs 65 kDa and so does not pass through the filter), the residual filtrate contained only the free UT fraction. The free KA concentration was below the limit of detection (< 0.01 mg/l) in most patients and so was not included in our analysis.

We conducted a literature review of *in vivo* and *in vitro* studies related to each of the 10 studied UTs in the CKD-REIN cohort to identify and select toxins with sufficient evidence of excretion via OAT1/OAT3 (Supplementary Table S1 and Table S2). IS, Kyn, PCS, IAA, and KA were selected, and free and total serum concentrations of these PBUTs were assessed. We considered the free fractions in the main analysis because only unbound UTs are toxic and are excreted by the OATs.

### Statistical Analyses

Continuous variables were reported as the median (interquartile range) or the mean (S), as appropriate. Categorical variables were quoted as absolute and relative values. First, we described the distribution of each selected PBUT concentration. The PBUT concentrations and other variables with a skewed distribution were log-transformed. We used the Wilcoxon rank-sum test to compare PBUT concentrations in patients with versus without furosemide prescriptions. We created a variable (denoted hereafter as ΣUTs_free_) to describe the sum of the free fraction concentrations of the PBUTs (IS, Kyn, PCS, and IAA) excreted by OAT1 and/or OAT3. This approach has been used in other studies of toxic effects.^39^ We then used scatter and box plots to describe the distribution of the log of each free PBUT concentration and ΣUTs_free_ by furosemide dose level category.

We next assessed the association between the PBUTs (as ΣUTs_free_ and for each individual PBUT) and furosemide status (i.e., furosemide prescription or not), dose category or dose level using a smooth function. We hypothesized that if furosemide contributes to PBUT accumulation in patients with CKD, the baseline values of ΣUTs_free_ will be higher in patients prescribed the OAT1/OAT3 inhibitor, furosemide. For ΣUTs_free_ and each free PBUT studied, crude and multiple linear models (fitted with the maximum likelihood) were used to assess the association between furosemide and the PBUTs at baseline. First, the furosemide dose level was treated as a categorical variable (0 mg, 10–40 mg, 60–120 mg, and > 120 mg). None of the patients had been prescribed a furosemide dose level < 10 mg or between 40 and 60 mg. The categories were chosen using quantiles of the prescribed dose level distribution. Next, the furosemide dose level was modelled as a continuous variable with natural splines and knots at 40, 80, 100, and 120 mg. We adjusted the data for the following clinically relevant factors identified in our literature review: age, sex, body mass index, smoking status, diabetes, a history of CV disease and acute kidney injury, eGFR (adjusted with natural splines, with 1 knot at 33 ml/min per 1.73 m^2^ and boundary knots at 8.3 and 89.7 ml/min per 1.73 m^2^), urine albumin-to-creatinine ratio (log), serum concentrations of CRP (log) and albumin, the prescription of potential OAT1/3-inhibiting drugs, and the number of other drugs prescribed. The assumptions of linearity, homoscedasticity, and normality of residuals for linear regression were assessed using residuals versus fitted value plots, scale-location plots, and Q-Q plots.

To deal with missing covariate data, we used the MICE package in R software (version 4.1.2) to perform multiple imputations with chained equations.^40^^,^^41^ The analysis required 24 imputed datasets to achieve replicable standard error estimates. The imputation model included all variables from the main analysis (cumulative free UTs, furosemide, and covariates). Linear regression models were generated for each dataset, and pooled regression coefficients were obtained according to Rubin’s rules. In a secondary analysis, we used the same models but with the total serum UT concentration as the dependent variable. As sensitivity analysis, we tested the interaction between furosemide dose level and eGFR and repeated the analysis with additional adjustment for uric acid. As a negative control, we performed the same analysis using hydrochlorothiazide as the dependent variable. The threshold for statistical significance was set to *P* < 0.05. All statistical analyses were performed with R software (version 4.1.2).

## Results

### The Patients’ Baseline Characteristics

At baseline, a total of 2342 patients were included (Table 1, Supplementary Figure S1). The median age was 68 years (interquartile range: 60–76), 1013 patients (66%) were men, 1211 (52%) had a history of CV disease, 955 (41%) had diabetes, and the mean eGFR was 35 ml/min per 1.73 m^2^ (Table 1). Of the patients, 799 (34%) had been prescribed furosemide. Relative to patients without a furosemide prescription, patients with a furosemide prescription were older and had a lower eGFR, greater frequencies of previous CV disease, diabetes and acute kidney injury, a higher CRP concentration, a lower hematocrit, and more drug prescriptions (Table 1).

**Table 1:** Patient characteristics at baseline, overall, and by furosemide prescription

Characteristic	Overall *N* = 2342	Furosemide prescription		Missing data (*n*, %)
		No *n* = 1543	Yes *n* = 799	
Sociodemographic variables				
Age (yrs), median [IQR]	68 [60–76]	67 [57–74]	71 [65–78]	0, 0%
Men	1550 (66%)	1013 (66%)	537 (67%)	0, 0%
Smoking status				14, 0.6%
Current smoker	287 (12%)	200 (13%)	87 (11%)	
Nonsmoker	947 (41%)	634 (41%)	313 (39%)	
Ex-smoker	1094 (47%)	700 (46%)	394 (50%)	
BMI (kg/m^2^), mean (SD)	28.8 (5.9)	27.6 (5.3)	31.0 (6.3)	44, 1.9%
History				
Cardiovascular	1211 (52%)	631 (41%)	580 (73%)	13, 0.6%
Atheromatous CV event	857 (37%)	441 (29%)	416 (53%)	26, 1.1%
Non-atheromatous CV event	741 (32%)	341 (22%)	400 (50%)	18, 0.8%
Diabetes	955 (41%)	490 (32%)	465 (58%)	5, 0.2%
Acute kidney injury	508 (23%)	290 (20%)	218 (29%)	169, 7.2%
Laboratory				
eGFR (ml/min per 1.73 m^2^), mean (SD)	35 (13)	37 (14)	30 (12)	15, 0.6%
Albumin-to-creatinine ratio (mg/g), median [IQR]	109 [22–492]	89 [19–454]	168 [30–591]	360, 15%
C-reactive protein (mg/l), median [IQR]	2.4 [1.1–5.1]	2.0 [1.0–4.4]	3.1 [1.5–6.6]	126, 5.4%
Serum albumin (g/l), mean (SD)	40.5 (4.2)	40.9 (4.1)	39.7 (4.2)	9, 0.4%
Hematocrit (%), mean (SD)	39.5 (4.8)	39.9 (4.8)	38.6 (4.8)	43, 1.8%
Potential OAT1- and/or OAT3-inhibiting drugs				
Number of prescribed OAT1 and/or OAT3 inhibitors, median [IQR]	1.0 [0.0–2.0]	1.0 [0.0–2.0]	1.0 [0.0–1.0]	0, 0%
Angiotensin II receptor blockers	1,031 (44%)	689 (45%)	342 (43%)	0, 0%
Proton pump inhibitors	761 (32%)	435 (28%)	326 (41%)	0, 0%
Thiazide and thiazide-like diuretics	501 (21%)	410 (27%)	91 (11%)	0, 0%
Number of other drugs prescribed, median [IQR]	7.0 [4.0–9.0]	5.0 [3.0–8.0]	9.0 [7.0–11.0]	0, 0%

BMI, body mass index; CV, cardiovascular; eGFR, estimated glomerular filtration rate; IQR, interquartile range; OAT, organic anion transporter.

### PBUTs and Furosemide Prescription

Apart from total IAA, the median serum free and total concentrations of all the studied toxins (ΣUTs_free_, IS, Kyn, PCS, and IAA) were significantly higher in patients with a furosemide prescription (Supplementary Table S3).

Among patients with a furosemide prescription and data on eGFR (*n* = 792), the proportion prescribed a 10 to 40 mg dose level ranged from 67% in the G2 to G3a group to 52% in the G4 to G5 group. In contrast, the proportion of patients receiving a high furosemide dose level increased as the eGFR decreased; 18% of the G2 to G3a group and 27% of the G4 to G5 group received 60 to 120 mg, and 15% of the G2 to G3a group and 21% of the G4 to G5 group received > 120 mg group (Supplementary Figure S2).

**Figure 2:**
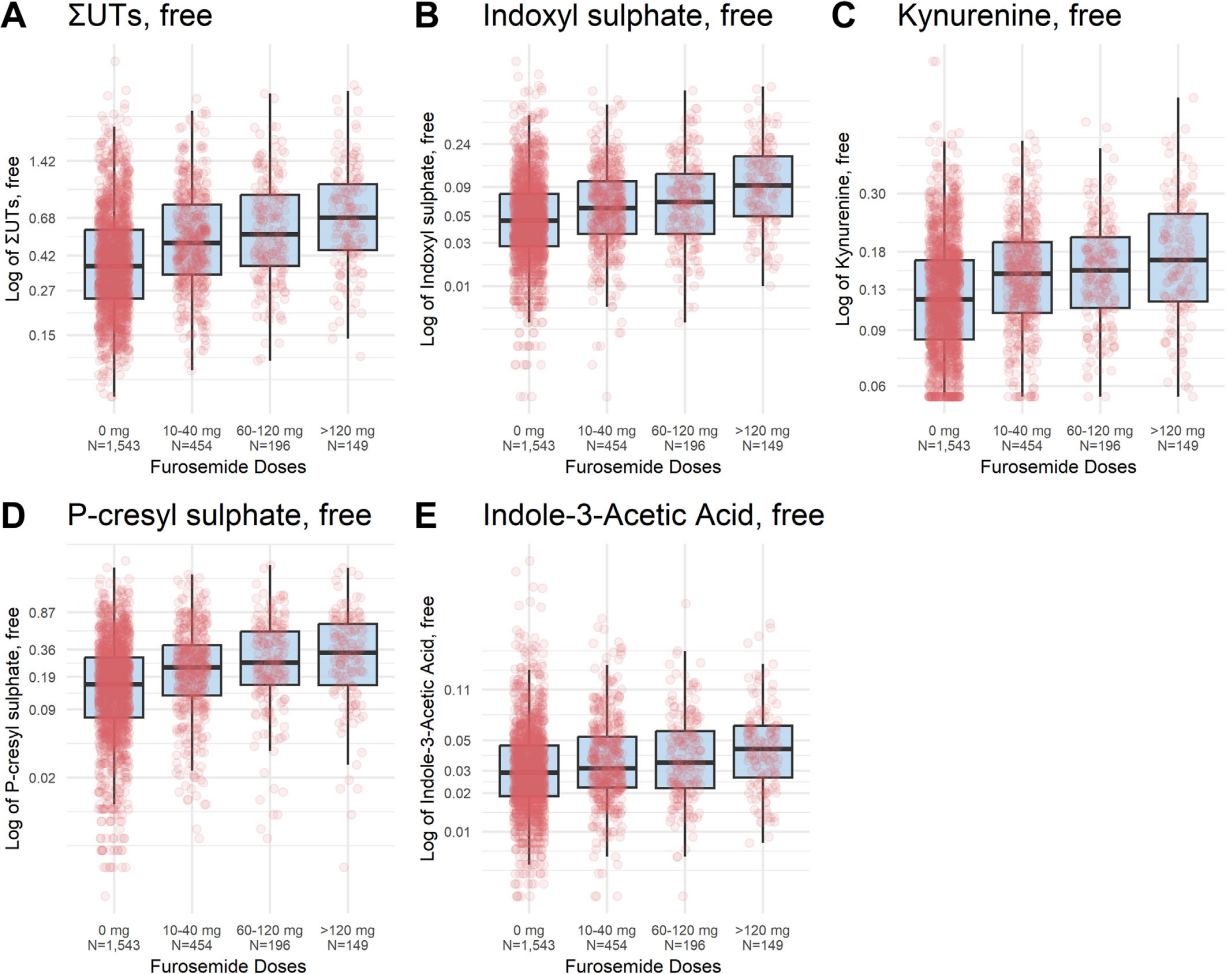
Distribution of free uremic toxins concentrations in patients with CKD, by furosemide dose level category. The ordinate axis represents the UT concentration (mg/l) corresponding to the 5th, 25th, 50th, 75th, and 95th quantiles. CKD, chronic kidney disease; UT, uremic toxin.

All the serum PBUT concentrations studied had a right-skewed distribution (Supplementary Figure S3). The logarithmic of serum free PBUT concentrations increased progressively across furosemide dose level categories (Figure 2).

### Associations Between PBUTs, Furosemide Status, and the Furosemide Dose Level Category

**Figure 3:**
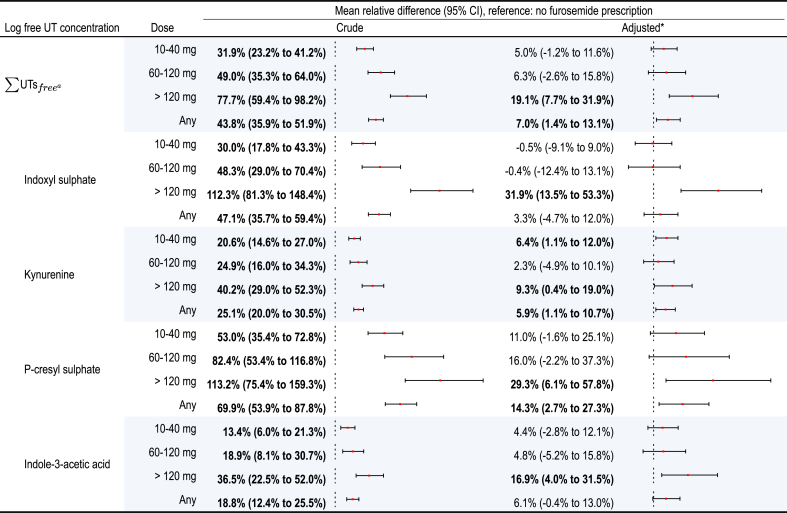
Mean relative difference in free uremic toxin concentration, as a function of the furosemide dose level category and furosemide status (reference: no furosemide prescription). 95% CIs that excluded 0% are given in bold type. ^a^The sum of free UTs, including the free fraction of indoxyl sulphate, kynurenine, p-cresyl sulphate and indole-3-acetic acid. ∗Adjusted for age, sex, the total number of concomitant prescription medications, the number of potential OAT1/3 inhibitors, the history of AKI and CV disease, serum CRP and albumin concentrations, diabetes, BMI, smoking status, uACR, and eGFR. AKI, acute kidney injury; BMI, body mass index; CI, confidence interval; CRP, C-reactive protein; CV, cardiovascular; eGFR, estimated glomerular filtration rate; OAT1/3, organic anion transporters 1 and 3; uACR, urinary albumin-to-creatinine ratio; UT, uremic toxin.

Simple linear regressions showed that apart from total IAA, all the PBUTs studied were associated with furosemide prescription status (i.e., prescription or not) and a higher furosemide dose level category (Figure 3).

After adjusting for age, sex, the total number of medications, potential OAT1/3 inhibitors, acute kidney injury, and CV disease history, serum CRP and albumin concentrations, diabetes, body mass index, smoking, urine albumin-to-creatinine ratio, and eGFR, a multiple linear regression showed a significant association between furosemide prescription and higher serum concentrations of ΣUTs_free_ (+7.0%; 95% confidence interval [CI]: 1.4%–13.1%), free Kyn (+5.9%; 95% CI: 1.1%–10.7%), and free PCS (+14.3%; 95% CI: 2.7%–27.3%) (Figure 3) in patients prescribed furosemide, compared with patients not prescribed furosemide. Similar but nonsignificant trends were observed for free IAA (+6.1%; 95%CI: −0.4% to 13.0%) (Figure 3). No significant associations were found for total UT concentrations (Supplementary Figure S4).

Patients prescribed > 120 mg of furosemide had significantly higher ΣUTs_free_ (+19.1%; 95% CI: 7.7%–31.9%), free IS (+31.9%; 95% CI: 13.5%–53.3%), free Kyn (+9.3%; 95% CI: 0.4%–19%), free PCS (+29.3%; 95% CI: 6.1%–57.8%), and free IAA (+16.9%; 95% CI: 4.0%–31.5%) concentrations, compared with those not prescribed furosemide (Figure 3). Furthermore, those receiving 10 to 40 mg had a higher free Kyn concentration (+6.4%; 95% CI: 1.1%–12.0%). In contrast, serum total KA concentrations were lower in patients prescribed > 120 mg (−8.2%; 95% CI: −15.6% to −0.1%) (Supplementary Figure S4).

### Associations Between PBUTs and the Furosemide Dose Level, After the Application of a Smooth Function

Crude linear regression with the use of a smooth function to model the relationships between the furosemide dose level and free UT concentrations revealed statistically significant, positive predicted differences at 20, 40, 60, 80, 120, and 250 mg, compared with patients without a furosemide prescription (Supplementary Figure S5). For IAA, the predicted differences were not significant at the 60 mg and 80 mg dose levels, compared with patients without a furosemide prescription.

**Figure 4:**
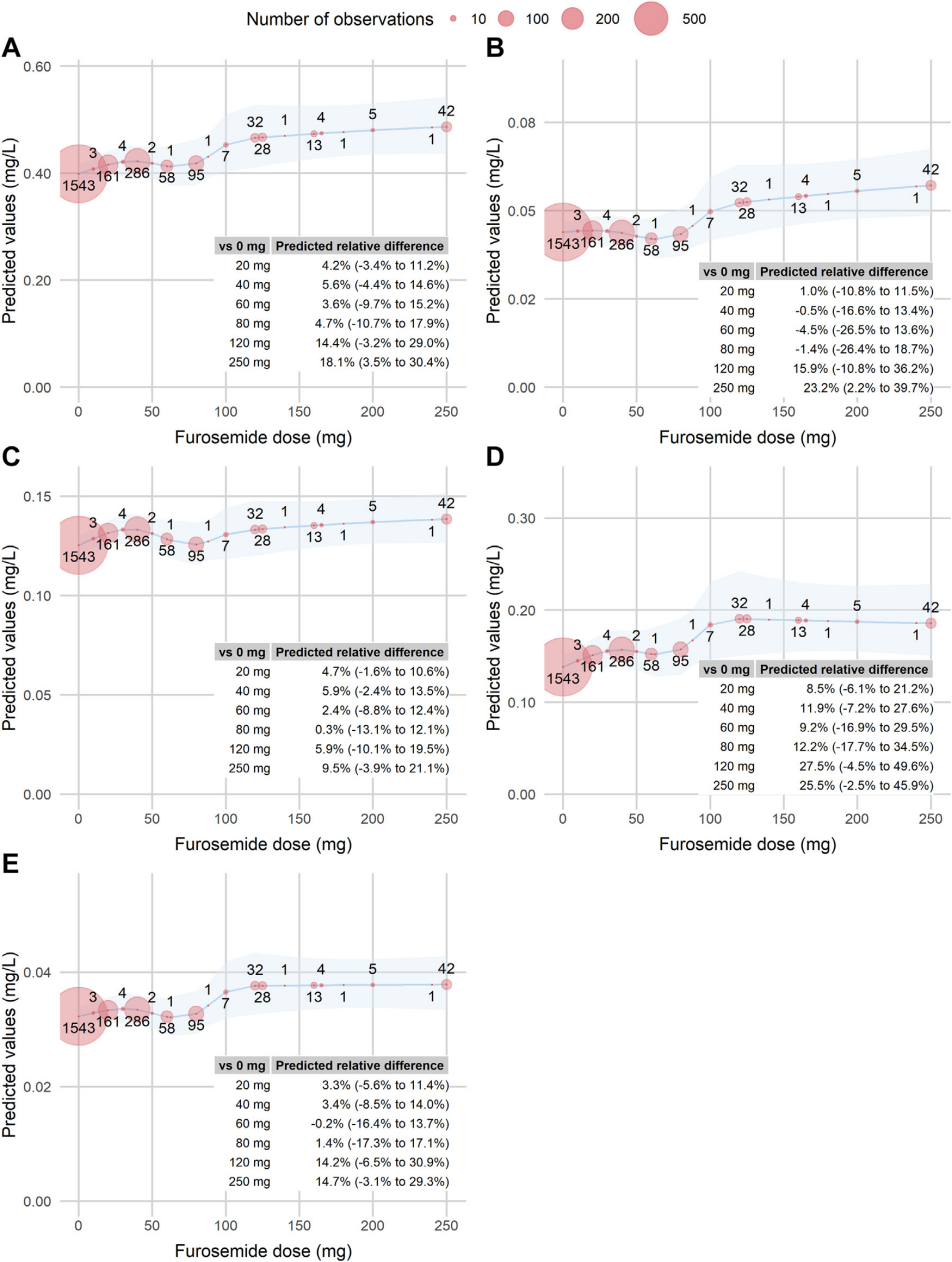
Predicted free uremic toxin concentrations as a smooth function of the furosemide dose level; adjusted model. The size of the red circles in the plots are proportional to the number of observations at each data point, with the exact number of observations indicated above each circle. A total of 54 observations are not shown (distributed between the dose levels of 290 and 1000 mg). For each plot, a table presents the predicted relative difference between no furosemide prescription and dose levels of 20, 40, 60, 80, 120, and 250 mg (the dose levels prescribed to > 30 patients). The furosemide dose level was modelled with natural splines with knots at 40 mg, 80 mg, 100 mg and 120 mg. The model was adjusted for age, sex, the total number of concomitant prescription medications, the number of potential OAT1/3 inhibitors, the history of AKI and CV disease, serum CRP and albumin levels, diabetes, BMI, smoking status, uACR, and eGFR. AKI, acute kidney injury; BMI, body mass index; CRP, c-reactive protein; CV, cardiovascular; eGFR, estimated glomerular filtration rate; OAT1/3, organic anion transporters 1 and 3; uACR, urinary albumin-to-creatinine ratio.

The adjusted models showed statistically significant predicted differences between patients without a furosemide prescription versus those with a dose level of 250 mg for ΣUTs_free_ (+18.1%; 95% CI: 3.5%–30.4%) and free IS (+20.9%; 95% CI: 2.2%–39.7%). For ΣUTs_free_ and each free UT, a steep increase between 80 and 100 mg was followed by a high plateau (Figure 4).

### Sensitivity Analysis

The relationship between the furosemide dose level category and the free IS concentration was more pronounced in patients with higher eGFR levels (> 45 ml/min per 1.73 m^2^); for the other PBUTs and their sum, the relationship was not influenced by the eGFR level (within the range observed in the study population, Supplementary Figure S6). Further adjustment for the uric acid concentration led to slight weakening of the associations between the furosemide dose level and PBUT concentrations (Supplementary Table S4). Lastly, as a negative control, we assessed the relationship between hydrochlorothiazide prescription and PBUT concentrations; as expected, no association was observed (Supplementary Figures S7 and S8).

## Discussion

In this cross-sectional analysis of a large cohort of patients with CKD stages G2 to G5, furosemide was associated with higher serum concentrations of free PBUTs. This finding suggests that *in vivo,* kidney tubular excretion of some PBUTs can be inhibited by the concomitant administration of a loop diuretic via competition at the kidney transporter level. The results of the present study show the following: (i) PBUT concentrations were significantly higher in patients prescribed furosemide than in those not prescribed furosemide, and (ii) this association was particularly strong in patients prescribed a furosemide dose level of > 120 mg. Furthermore, this association was independent of other risk factors, such as the eGFR.

The hematocrit was not higher in patients prescribed furosemide than in those not prescribed furosemide; this lack of a difference was possibly because of the lower eGFR observed in the former group. Furthermore, no published studies have found that diuretics cause a long-term increase in the hematocrit; any increase is observed only shortly after the administration of the loop diuretic (lasting up to 3 hours).^42^^,^^43^

We evaluated the potential interaction between a drug and PBUT concentrations through competition for kidney transporters. We extended previous findings about drugs that are widely prescribed to patients with CKD. For example, an *in vivo* study in rats demonstrated that the administration of ciprofloxacin decreased the renal clearance of IS.^27^ Importantly, the results of an *in vitro* study of a proximal tubule cell line suggested that in patients with CKD, IS, PCS, and KA might compete with commonly prescribed drugs for OAT1-mediated secretion.^28^ At low drug concentrations, competing inhibition was primarily influenced by UTs. However, significant inhibition occurred at higher concentrations of furosemide, valsartan, and losartan (within their therapeutic windows).^28^ In a study that included kidney transplant patients, serum PCS concentrations were significantly higher in patients prescribed at least 1 OAT inhibitor (*n* = 311) than in patients not prescribed an OAT inhibitor (*n* = 92), after adjustment for age, eGFR, the serum albumin concentration, and time since transplantation.^29^ Lastly, a CKD-REIN study showed that after adjustments for baseline comorbidities, the number of coprescribed drugs, and laboratory variables (including the eGFR), proton-pump inhibitor prescription was significantly associated with elevated serum concentrations of free and total IS, free and total p-cresyl glucuronide, and phenylacetylglutamine.^30^ The results of all these studies suggest that some drugs compete with PBUTs for OATs.

We found that the associations between the furosemide dose level and the free UT concentrations were slightly weaker after additional adjustment for uric acid. The inhibition of urate secretion by hOAT1 and hOAT3 has previously been investigated in 95 healthy volunteers; significantly lower excretion of urate was observed after torasemide administration.^44^ Furthermore, furosemide was associated with a significantly higher serum urate concentrations in humans.^45^^,^^46^ However, the association between hyperuricemia and its clinical consequences remains subject to debate.^47^ Although uric acid inhibits OAT1 and OAT3,^48^ its kinetic parameters (K_m_ ≈ 943 ± 84 μM for hOAT1^49^ and IC_50_ ≈ 255 ± 34 μM for hOAT3-mediated uptake of estrone sulphate^50^) are higher than those of the PBUTs considered in our study.

PBUT clearance in the kidney is controlled by tubular secretion. In the proximal tubule, bound solute fractions shift to free factions before excretion by OATs 1 and 3. In our study, we observed higher concentrations of free PBUTs and no differences in total PBUT concentrations at dose levels exceeding 120 mg. In patients with CKD, albumin binding capacity is significantly below the reference range for healthy individuals.^51^ Several studies have reported that the protein-bound proportions of various PBUTs are lower in patients with CKD than in healthy individuals.^10^^,^^51^, ^52^, ^53^ This lower proportion is thought to result from the saturation of albumin binding sites by PBUTs (i.e., competition among PBUTs for albumin binding) and posttranslational modifications of albumin (e.g., oxidation, glycosylation, and carbamylation).^51^ Our results support the hypothesis that furosemide competes for binding sites on albumin.^54^^,^^55^ We could also consider an indirect effect of furosemide. For example, high dose levels of furosemide may induce an alkaline pH,^56^ which could alter albumin’s conformation (the protein is known to undergo pH-dependent structural transitions)^57^ and thus reduce its binding affinity. This change might contribute to the observed increase in the free fraction of PBUTs.

Better knowledge of the competition between drugs and PBUTs might help to understand the mechanisms that lead to the accumulation of PBUTs in patients with CKD. Although many *in vitro* studies have identified medications as OAT1/OAT3 inhibitors, the concentrations are often outside the therapeutic range. André *et al.*^29^ reported on a number of potential inhibitors at therapeutic dose levels; hydrochlorothiazide (not associated with PBUTs in our study) was not among them. However, several medications (including proton-pump inhibitors, other diuretics, nonsteroidal antiinflammatory drugs, antivirals, and antibiotics) exhibited a competitive effect.^23^^,^^25^^,^^26^ It is therefore important to consider this potential interaction when prescribing new drugs to patients with polypharmacy or when reviewing current prescriptions. As mentioned above, polypharmacy is common in patients with CKD. Notably, loop diuretics are often prescribed to patients with CKD, to control hypertension and hypervolemia, especially when the eGFR is < 30 ml/min per 1.73 m^2^.^58^ Furosemide use might induce various adverse reactions, such as hypotension, hyponatremia, and hypokalemia. Here, we described a potential new adverse drug reaction: the accumulation of certain PBUTs. In the present study, patients with a bumetanide prescription had higher median UT concentrations than patients without a bumetanide prescription. However, because of the small number of bumetanide-treated patients (*n* = 25), we cannot say for sure whether these results are independent of kidney function and other risk factors. One can nevertheless hypothesize that bumetanide may be a viable, readily available alternative to furosemide. Therapeutic concentrations of bumetanide inhibit OAT3 but not OAT1^29^; relative to furosemide, bumetanide use might result in weaker drug-PBUT competition, less PBUT retention, and potential improvements in symptoms and clinically relevant outcomes. However, the results of retrospective studies of loop diuretic use have been inconclusive with regard to which specific loop diuretic is most effective in terms of long-term clinical outcomes and symptom management; this was probably because of confounding by indication.^59^, ^60^, ^61^, ^62^, ^63^, ^64^ In contrast, the loop diuretic, torasemide is probably not an option because it inhibits both OAT1 and OAT3 and reduces urate excretion.^44^ Further prospective studies of furosemide versus bumetanide are essential for determining whether one or the other has more beneficial effects on symptoms and clinically relevant outcomes (potentially mediated by differences in PBUT clearance) in patients with CKD.

The results of the present study suggested the presence of a threshold effect for furosemide-PBUT competition at dose levels above 120 mg. It is important to note that the association between furosemide status and the concentrations of PBUTs (particularly IS and IAA) would have been masked if we had not taken account of the furosemide dose level and thus evidenced a threshold effect. In contrast, PCS appeared to exhibit a dose-response effect. One possible explanation for these different effects might relate to the kinetics of each PBUT’s excretion via OAT1 and/or OAT3. Molecules with a low inhibitory constant (K_i_), Michaelis constant (K_M_) or half-maximal inhibitory concentration (IC_50_) exhibit greater affinity for the transporter in question and are excreted more readily. OAT1 and OAT3 have similar affinities and inhibition potencies for furosemide, as indicated by K_M_ values of 38.9 μM and 21.5 μM, IC_50_ values of 18 μM and 7.43 μM, and K_i_ values of 11.4 μM and 5.41 μM, respectively.^31^, ^32^, ^33^, ^34^ IS, PCS, IAA, and Kyn have higher K_i_, K_M_, or IC_50_ values than furosemide and might thus compete with the drug for their excretion (Table S1). Notably, PCS appears to have higher K_i_, K_M_, and IC_50_ values than the other PBUTs studied, which may explain the observed differences in dose-response effects.

Our study had several strengths. First, we assessed a large number of patients with a confirmed diagnosis of CKD and who had been recruited through a nationally representative sample of nephrology outpatient facilities. Second, our detailed survey enabled us to identify all the furosemide dose levels prescribed and gave us information on potential confounders (e.g., eGFR, comorbidities, and other potential OAT1/3 inhibitors prescribed). Third, free and total UT concentrations were measured in the same central laboratory, using a robust, validated, ultrahigh-performance liquid chromatography tandem mass spectrometry technique. Lastly, our analysis of the free UT fractions enabled us to highlight the association between furosemide and PBUT concentrations; had we focused solely on the total UT concentration, this association would not have been observed.

Our study also had limitations. First, the cross-sectional design precludes us from assessing the temporal sequence of the relationship between serum PBUT concentrations and furosemide. However, given that previous published data indicate that the inhibition constant (Ki) is higher for PBUTs than for furosemide, it is more plausible that furosemide dose level influences PBUT concentration than vice versa. Second, although we adjusted our models for several factors that potentially influence furosemide prescription and UTs, we cannot rule out residual confounding. For example, we do not have data on proximal tubular damage, which could contribute to the impaired excretion of PBUTs. Third, our definition of furosemide exposure was based on prescriptions, meaning we could not confirm actual medication intake. Lastly, though the variable timing of blood sample collection may have introduced some measurement variability in UT concentrations, this effect was likely limited because of the chronic (rather than acute) administration of furosemide. Despite these limitations, our findings might provide a basis for future longitudinal analyses and might be useful for understanding drug-PBUT competition.

In conclusion, the results of the present study showed that the commonly prescribed drug furosemide was associated with higher serum PBUT concentrations (including ΣUTs_free_, IS, PCS, IAA, and Kyn) in patients with CKD and thus highlights a potential adverse reaction to furosemide. The balance between furosemide’s beneficial effects and adverse reactions must always be considered. In view of the association between PBUTs and CKD-related morbidity and mortality, our findings provide valuable insights into the factors that might influence serum PBUT concentrations in patients with CKD.

## Appendix

### List of the members of the CKD-REIN Study Group

Natalia Alencar De Pinho, Dorothée Cannet, Christian Combe, Denis Fouque, Luc Frimat, Aghilès Hamroun, Yves-Edouard Herpe, Christian Jacquelinet, Maurice Laville, Sophie Liabeuf, Ziad A. Massy, Pascal Morel, Christophe Pascal, Roberto Pecoits-Filho, Joost Schanstra, Bénédicte Stengel, Céline Lange, Oriane Lambert, and Marie Metzger.

## Disclosure

NAP coordinates the CKD-REIN cohort study, and received funding as indicated in the section “Funding”. ZAM reports having received grants for CKD-REIN and other research projects from Amgen, Baxter, Fresenius Medical Care, GlaxoSmithKline, Merck Sharp & Dohme-Chibret, Sanofi- Genzyme, Lilly, Otsuka, AstraZeneca, Vifor and the French government, as well as fees and grants to charities from AstraZeneca, Boehringer Ingelheim, and GlaxoSmithKline. DF reports honoraria and travel support from Astellas, AstraZeneca, DrSchar, FreseniusKabi, GSK, and Lilly. All the other authors declared no competing interests.

## Supplementary Material

Supplementary File (PDF) **Supplementary Acknowledgments. Supplementary References. Figure S1.** Study flow chart. **Figure S2.** Furosemide dose level prescribed, by eGFR level. **Figure S3.** Distribution of protein-bound uremic toxin concentration before and after log transformation. **Figure S4.** Mean relative difference in total uremic toxin concentration, as a function of furosemide dose level category and furosemide status (reference: no furosemide prescription). **Figure S5.** Predicted protein-bound uremic toxin concentrations as a smooth function of furosemide dose level; crude model. **Figure S6.** Predicted free uremic toxin concentrations as a function of furosemide dose level category, stratified by different eGFR levels. **Figure S7.** Mean relative difference in free uremic toxin levels, as a function of the hydrochlorothiazide dose level category (reference: no hydrochlorothiazide prescription). **Figure S8.** Mean relative difference in total uremic toxin levels, as a function of the hydrochlorothiazide dose level category (reference: no hydrochlorothiazide prescription). **Table S1.** Pharmacokinetics of protein-bound uremic toxin transport by OAT1 and/or OAT3, based on the results of *in vitro* studies. **Table S2.** Transport of protein-bound uremic toxins by OAT1 and/or OAT3, based on the results of *in vivo* studies. **Table S3.** Median [interquartile range] protein-bound uremic toxin concentrations at baseline, overall and by furosemide prescription status. **Table S4.** Mean relative difference in free uremic toxin levels, as a function of the furosemide dose level category and furosemide status and after further adjustment for uric acid. **STROBE Checklist.**

## Footnotes

Supplementary File (PDF)

**Supplementary Acknowledgments.**

**Supplementary References.**
**Figure S1.** Study flow chart. **Figure S2.** Furosemide dose level prescribed, by eGFR level. **Figure S3.** Distribution of protein-bound uremic toxin concentration before and after log transformation. **Figure S4.** Mean relative difference in total uremic toxin concentration, as a function of furosemide dose level category and furosemide status (reference: no furosemide prescription). **Figure S5.** Predicted protein-bound uremic toxin concentrations as a smooth function of furosemide dose level; crude model. **Figure S6.** Predicted free uremic toxin concentrations as a function of furosemide dose level category, stratified by different eGFR levels. **Figure S7.** Mean relative difference in free uremic toxin levels, as a function of the hydrochlorothiazide dose level category (reference: no hydrochlorothiazide prescription). **Figure S8.** Mean relative difference in total uremic toxin levels, as a function of the hydrochlorothiazide dose level category (reference: no hydrochlorothiazide prescription). **Table S1.** Pharmacokinetics of protein-bound uremic toxin transport by OAT1 and/or OAT3, based on the results of *in vitro* studies. **Table S2.** Transport of protein-bound uremic toxins by OAT1 and/or OAT3, based on the results of *in vivo* studies. **Table S3.** Median [interquartile range] protein-bound uremic toxin concentrations at baseline, overall and by furosemide prescription status. **Table S4.** Mean relative difference in free uremic toxin levels, as a function of the furosemide dose level category and furosemide status and after further adjustment for uric acid.
**STROBE Checklist.**
