# Supplementary material for: Furosemide and Serum Protein-Bound Uremic Toxin Concentrations in Patients With CKD
Source: Kidney Int Rep. 2025 May 2;10(7):2165–77. doi: 10.1016/j.ekir.2025.04.040 (PMC12266279; doi:10.1016/j.ekir.2025.04.040)
Supplement: Supplementary File (PDF) — Supplementary Acknowledgments. Supplementary References. Figure S1. Study flow chart. Figure S2. Furosemide dose level prescribed, by eGFR level. Figure S3. Distribution of protein-bound uremic toxin concentration before and after log transformation. Figure S4. Mean relative difference in total uremic toxin concentration, as a function of furosemide dose level category and furosemide status (reference: no furosemide prescription). Figure S5. Predicted protein-bound uremic toxin concentrations as a smooth function of furosemide dose level; crude model. Figure S6. Predicted free uremic toxin concentrations as a function of furosemide dose level category, stratified by different eGFR levels. Figure S7. Mean relative difference in free uremic toxin levels, as a function of the hydrochlorothiazide dose level category (reference: no hydrochlorothiazide prescription). Figure S8. Mean relative difference in total uremic toxin levels, as a function of the hydrochlorothiazide dose level category (reference: no hydrochlorothiazide prescription). Table S1. Pharmacokinetics of protein-bound uremic toxin transport by OAT1 and/or OAT3, based on the results of in vitro studies. Table S2. Transport of protein-bound uremic toxins by OAT1 and/or OAT3, based on the results of in vivo studies. Table S3. Median [interquartile range] protein-bound uremic toxin concentrations at baseline, overall and by furosemide prescription status. Table S4. Mean relative difference in free uremic toxin levels, as a function of the furosemide dose level category and furosemide status and after further adjustment for uric acid. STROBE Checklist. [file mmc1.pdf]

Supplementary material

Figure S1. Study flow chart

Table S1. Pharmacokinetics of protein-bound uraemic toxin transport by OAT1 and/or OAT3, based on the results of *in vitro* studies.

Table S2. Transport of protein-bound uraemic toxins by OAT1 and/or OAT3, based on the results of *in vivo* studies.

Table S3. Median [interquartile range] protein-bound uraemic toxin concentrations at baseline, overall and by furosemide prescription status.

Figure S2. Furosemide dose level prescribed, by eGFR level.

Figure S3. Distribution of protein-bound uraemic toxin concentration before and after log transformation.

Figure S4. Mean relative difference in total uraemic toxin concentration, as a function of furosemide dose level category and furosemide status (reference: no furosemide prescription).

Figure S5. Predicted protein-bound uraemic toxin concentrations as a smooth function of furosemide dose level, crude model

Figure S6. Predicted free uraemic toxin concentrations as a function of furosemide dose level category, stratified by different eGFR levels.

Table S4. Mean relative difference in free uraemic toxin levels, as a function of the furosemide dose level category and furosemide status and after further adjustment for uric acid.

Figure S7. Mean relative difference in free uraemic toxin levels, as a function of the hydrochlorothiazide dose level category (reference: no hydrochlorothiazide prescription).

Figure S8. Mean relative difference in total uraemic toxin levels, as a function of the hydrochlorothiazide dose level category (reference: no hydrochlorothiazide prescription).

Supplementary Acknowledgments

STROBE Checklist

Supplementary References

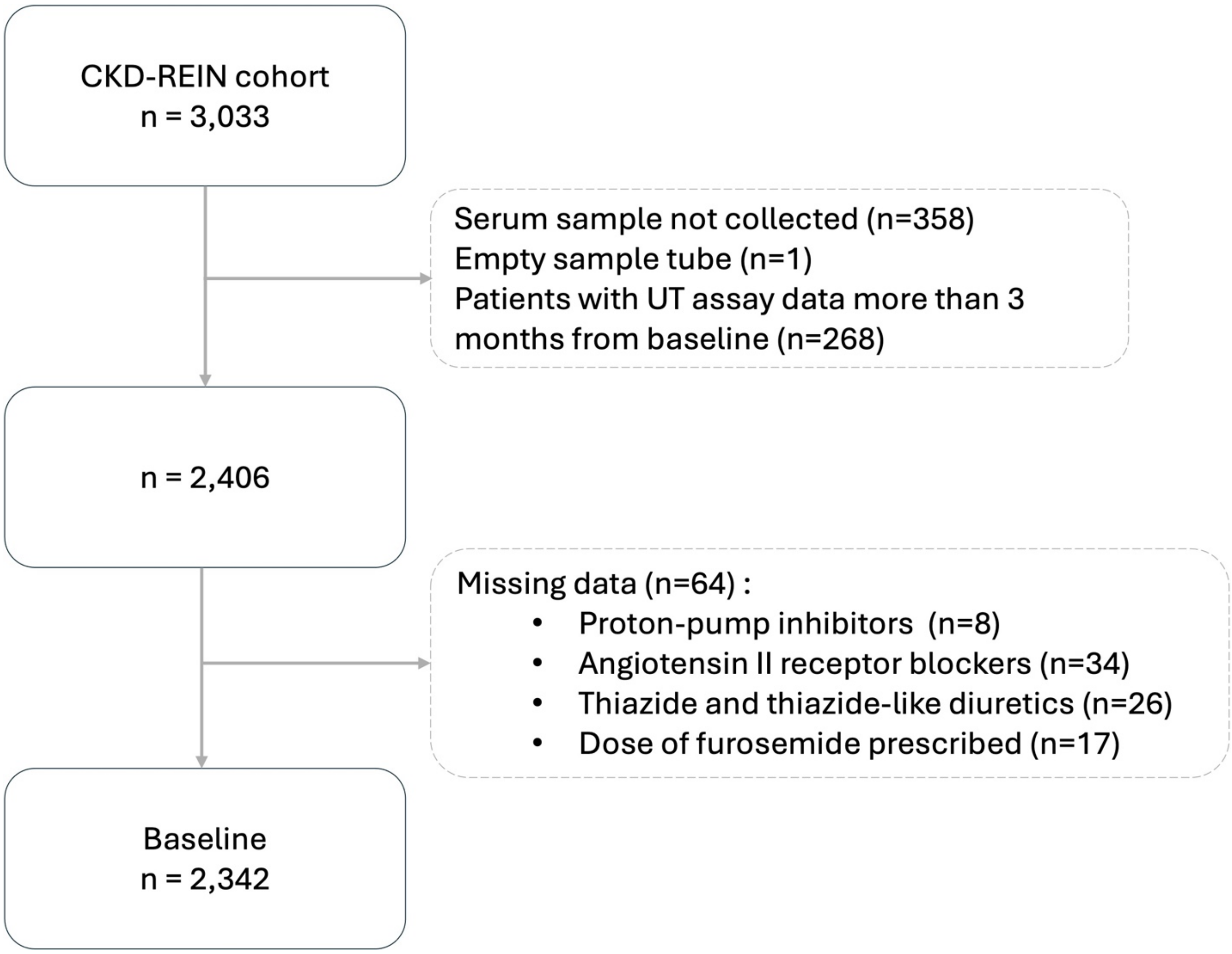

Figure S1. Study flow chart

Abbreviations: UT, uraemic toxin

Table S1. Pharmacokinetics of protein-bound uraemic toxin transport by OAT1 and/or OAT3, based on the results of *in vitro* studies.

Uraemic Toxins	OAT inhibitors	Type of OAT	Km (μM)		Ki (μM)		IC50 (μM)		Reference
			OAT1	OAT3	OAT1	OAT3	OAT1	OAT3	
**Hippurate**	IxS	rOAT						11.9	S1
	6-CF	hOAT					31	41	11
	PAH/pravastatin/ PCG	rOAT	27.5		27.5	18.6			S2
		hOAT	23.5		18.8	30.8			
	PAH	hOAT					20		12
	PAH/PCG	mkOAT	12.2						S3
	fluorescein	hOAT					5	22	S4
	M7	hOAT					162	87.4	S5
	M8-1							39.4	
	M8-2							24.1	
**Indole acetate**	IxS	rOAT3						509	S1
	6-CF	hOAT					140		11
	PAH/pravastatin/PCG	rOAT	47.1		48.5	582			S2
		hOAT	14		21	491			
	PAH	hOAT					83		12
	PAH/PCG	mkOAT	23.6						S3
	M7	hOAT					197	404	S5
	M8-1							181	
	M8-2							173	
**Indoxyl sulphate**	Pbe/ PAH/PCG/cimetidine	rOAT		158					S1
	6-CF	hOAT					110	270	11
	6-CF	mOAT					18		13
	PAH/pravastatin/PCG	rOAT	17.7	174	25	138			S2
		hOAT	20.5	263	13.2	183			
	PAH	hOAT					83		12
	PAH/PCG	mkOAT	32.9						S3
	PAH/ES	hOAT			22.7	168.7	47.3	80.4	S6
		rOAT		136.7					
	PAH/ES	rOAT			34.2	74.4			S7
	-	hOAT			Transport by OAT1				11
	fluorescein	hOAT					25	83	S4
	Pbe	hOAT			Transport by OAT1/3				12

	M7						200	222	
	M8-1	hOAT						161	S5
	M8-2							78.3	
**Kynurenate**	fluorescein	hOAT					6	6	S4
	6-CF	hOAT					34	23	11
	PAH/6-CF	mOAT					34	8	S8
	PAH/EF	hOAT	5.06	4.86			12.9	7.76	S9
**Kynurenine**	fluorescein	hOAT					65	219	S4
	6-CF	mOAT					12		13
**p-cresyl sulphate**	fluorescein	hOAT					79	112	S4
	6-CF	hOAT					210	200	11
	PAH/ES/ Pbe	hOAT	128	Not observed at 5mM			690	485	14
	-	hOAT			Transport by OAT1				11
	-	rOAT	231.6						16
	Pbe/PCG/PAH/ EF	hOAT			Transport by OAT1/3, + OAT3				
	IS/IA/CMPF/HA				IS/CMPF: 80% inhibition, HA: 50% inhibition				
	Pbe	hOAT			OAT1/3 transporter				12
**p-cresyl glucorinide**	fluorescein	hOAT					2650	588	S4
**CMPF**	IxS	rOAT						4.01	S1
	6-CF	hOAT					79	28	11
	PAH/pravastatin/PCG	rOAT	154	10.9	103	27.9			S2
		hOAT	141	26.5	247	27.9			
	PAH/PCG	mkOAT	85.3	18.6					S3
	PAH	rOAT	194		91				S11
	M7						187	19.2	
	M8-1	hOAT						8.53	S5
	M8-2							6.75	
	PCG	rOAT		6.43					19

OAT3 inhibitors: cimetidine, ES, M8-1/M8-2, pravastatin, PCG

OAT1 inhibitors: PAH

OAT1 and OAT3 inhibitors; IxS, M7, Pbe, 6-CF, fluorescein

No *in vitro* data were found for trimethylamine N-oxide (TMAO) and phenylacetylglutamine (PAG).

Km: the Michaelis constant, the substrate concentration at which the reaction rate is 50% of the V_{max} (maximum velocity). A low Km value suggests a high affinity for the transporter.
Ki: the inhibitory constant, the concentration of an inhibitor required to reduce the activity of a receptor by 50%.
IC50: Inhibitory concentration 50%, the concentration of an inhibitor needed to inhibit a biological process or response by 50%.
Abbreviations: 6-CF, 6-carboxyfluorescein; CMPF, 3-carboxy-4-methyl-5-propyl-2-furanpropionate; ES, estrone sulphate; hOAT, human OAT; IxS, indoxyl sulphate; M8-1/M8-2, morinidazole glucuronide conjugates; M7, morinidazole sulphate conjugate; mOAT, mouse OAT; mkOAT, monkey OAT; OAT, organic anion transporter; PAH, p-aminohippurate; Pbe, probenecid; PCG, benzylpenicillin; rOAT, rat OAT.

Table S2. Transport of protein-bound uraemic toxins by OAT1 and/or OAT3, based on the results of *in vivo* studies.

**Uraemic Toxins**		**Comparison**	**Changes**			**Reference**
			OAT1	OAT3	OAT1/OAT3	
**Hippurate**	Mice	KO vs WT	NS	NS		15
		OAT3 KO + Pbe vs OAT3 KO	NS	x		
	Humans	Pbe			Elevated in plasma/NS in urine	16
	Rats	PAH/PCG	75%	10%	85%	20
	Mice	KO vs WT		NS		18
**Indole acetate**	Mice	KO vs WT	NS	Elevated in plasma		15
		OAT3 KO + Pbe vs OAT3 KO	NS	x		
	Mice	KO vs WT		Elevated in plasma		18
	Mice	KO vs WT	NS	NS		17
	Humans	Pbe vs untreated			Elevated in plasma	
	Humans	Pbe vs untreated			Elevated in plasma/low in urine	16
	Mice	KO vs WT	NS	Elevated in plasma		
	Rats	PAH/PCG	90%	10%	100%	20
	Humans	Pbe vs untreated			Elevated in plasma	S12
**Indoxyl sulphate**	Mice	KO vs WT	Elevated in plasma			S13
	Humans	Pbe vs untreated			Elevated in plasma/low in urine	
	Mice	KO vs WT	Elevated in plasma			13
	Mice	KO vs WT		Elevated in plasma		15
		OAT3 KO + Pbe vs OAT3 KO	NS	x		
	Mice	KO vs WT		Elevated in plasma		18
	Mice	KO vs WT	Elevated in plasma	Elevated in plasma		17
	Humans	Pbe vs untreated			Elevated in plasma	
	Humans	Pbe vs untreated			Elevated in plasma/low in urine	16

	Mice	KO vs WT	Elevated in plasma	Elevated in plasma		
	Mice	Pbe /PAH/PCG/cimetidine		Inhibitor	Inhibitor	S1
	Rats	PAH/PCG	52%	45%	97%	20
	Rats	Pbe<br>Quinapril		 Inhibitor (50%)	Inhibitor (85%) 	S14
	Rats	Pbe			Elevated in plasma	S15
**Kynurenate**	Mice	KO vs WT<br>OAT3 KO + Pbe vs OAT3 KO	Elevated in plasma<br>Elevated in plasma	NS<br>x		15
	Mice<br>Humans	KO vs WT<br>Pbe vs untreated	Elevated in plasma 	NS 	 Elevated in plasma	17
	Humans	Pbe vs untreated			Elevated in plasma/NS in urine	16
	Humans	Pbe vs untreated			Elevated in plasma	S12
**Kynurenine**	Mice	KO vs WT	Elevated in plasma			13
	Mice	KO vs WT<br>OAT3 KO + Pbe vs OAT3 KO	NS<br>Elevated in plasma	Elevated in plasma<br>x		15
	Mice<br>Humans	KO vs WT<br>Pbe vs untreated	Elevated in plasma 	Elevated in plasma (p<0.1) 	 Elevated in plasma	17
	Humans<br>Mice	Pbe vs untreated<br>KO vs WT	 Elevated in plasma	 Elevated in plasma	Elevated in plasma/low in urine 	16
**p-cresyl sulphate**	Mice	KO vs WT	Elevated in plasma			S13
	Mice	KO vs WT<br>OAT3 KO + Pbe vs OAT3 KO	NS<br>Elevated in plasma	Elevated in plasma<br>x		15
	Mice	KO vs WT		Elevated in plasma		18
	Humans<br>Mice	Pbe vs untreated<br>KO vs WT	 Elevated in plasma	 Elevated in plasma	Elevated in plasma/low in urine 	16
	Humans	Pbe vs untreated			Elevated in plasma	S12
**p-cresyl glucorinide**	Mice	KO vs WT		Elevated in plasma		18
	Mice	KO vs WT	Elevated in plasma	Elevated in plasma		17
	Humans<br>Mice	Pbe vs untreated<br>KO vs WT	 Elevated in plasma	 Elevated in plasma	Elevated in plasma/low in urine 	16
**CMPF**	Mice	KO vs WT	Elevated in plasma			S13
	Mice	KO vs WT<br>OAT3 KO + Pbe vs OAT3 KO	NS<br>NS	Elevated in plasma (p <0.1) 		15
	Mice	KO vs WT		Elevated in plasma (p<0.1)		18
	Rats	PAH/PCG	35%	65%	100%	20

	Mice	Pbe vs untreated			Down in myocardial cells	S16
TMAO	Mice	KO vs WT<br>OAT3 KO + Pbe vs OAT3 KO	NS<br>NS	Elevated in plasma		15
	Mice	KO vs WT		Elevated in plasma		18
	CV Humans	Pbe vs untreated			NS	S17
PAG	Mice	KO vs WT<br>OAT3 KO + Pbe vs OAT3 KO	NS<br>NS	NS		15
	Humans	Pbe vs untreated			Elevated in plasma/NS in urine	16

The reported % values correspond to the percentage uptake.

OAT3 inhibitors: cimetidine, quinapril, PCG

OAT1 inhibitors: PAH

OAT1 and OAT3 inhibitors: Pbe

Abbreviations: CMPF, 3-carboxy-4-methyl-5-propyl-2-furanpropionate; CV, cardiovascular disease; KO, knockout; NS, non-significant; OAT, organic anion transporter; PAG, phenylacetylglutamine; PAH, p-aminohippurate; Pbe, probenecid; PCG, benzylpenicillin; TMAO, trimethylamine N-oxide (TMAO); WT, wild type.

Table S3. Median [interquartile range] protein-bound uraemic toxin concentrations at baseline, overall and by furosemide prescription status.

Uraemic toxins (mg/L)	Overall n=2,342	Furosemide prescription		p-value[a]
		No n=1,543	Yes n=799	
**ΣUTs_{free}[b]**	0.42 [0.27; 0.68]	0.37 [0.25; 0.59]	0.53 [0.36; 0.89]	<0.001
Indoxyl sulphate, free	0.05 [0.03; 0.09]	0.05 [0.03; 0.08]	0.07 [0.04; 0.12]	<0.001
Kynurenine, free	0.13 [0.09; 0.18]	0.12 [0.08; 0.17]	0.15 [0.11; 0.21]	<0.001
P-cresyl sulphate, free	0.19 [0.09; 0.36]	0.16 [0.07; 0.30]	0.26 [0.13; 0.50]	<0.001
Indole-3-acetic acid, free	0.032 [0.023; 0.050]	0.031 [0.022; 0.047]	0.037 [0.025; 0.057]	<0.001
**Sum of total UTs[c]**	20 [12; 32]	18 [11; 29]	24 [15; 37]	<0.001
Indoxyl sulphate, total	4.1 [2.5; 6.9]	3.8 [2.3; 6.2]	4.8 [2.8; 8.1]	<0.001
Kynurenine, total	1.19 [0.91; 1.60]	1.15 [0.88; 1.56]	1.25 [0.96; 1.68]	<0.001
P-cresyl sulphate, total	13 [7; 22]	12 [6; 20]	17 [9; 27]	<0.001
Indole-3-acetic acid, total	0.58 [0.41; 0.84]	0.58 [0.41; 0.83]	0.59 [0.41; 0.88]	0.4
Kynurenic acid, total	0.030 [0.021; 0.045]	0.029 [0.020; 0.043]	0.033 [0.023; 0.049]	<0.001

[a] Wilcoxon rank sum test, [b] the sum of free UTs, including the free fraction of indoxyl sulphate, kynurenine, p-cresyl sulphate and indole-3-acetic acid, [c] the sum of total UTs included the total fraction of indoxyl sulphate, kynurenine, p-cresyl sulphate, indole-3-acetic acid and kynurenic acid.
Abbreviations: UT, uraemic toxin

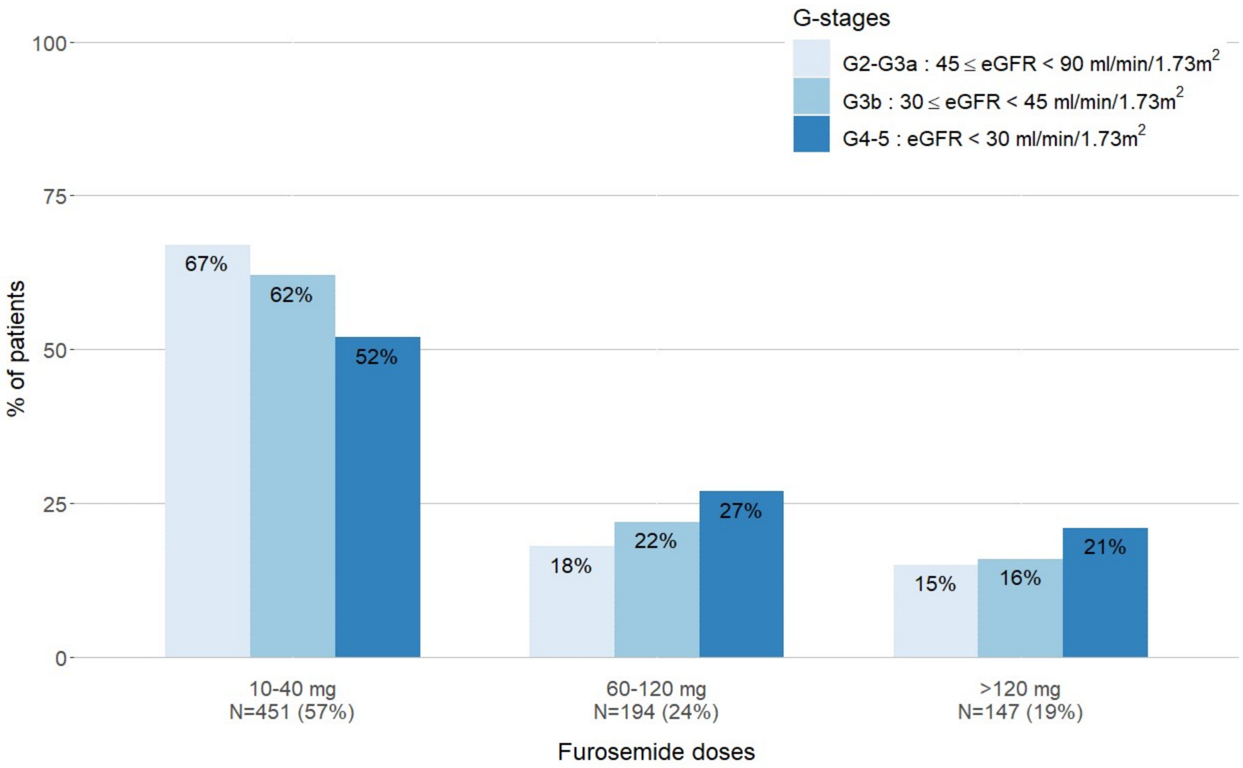

Figure S2. Furosemide dose level prescribed, by eGFR level.
Seven patients with missing eGFR data were excluded, leaving 792 patients with a furosemide prescription in this analysis.
Abbreviations: eGFR, estimated glomerular filtration rate

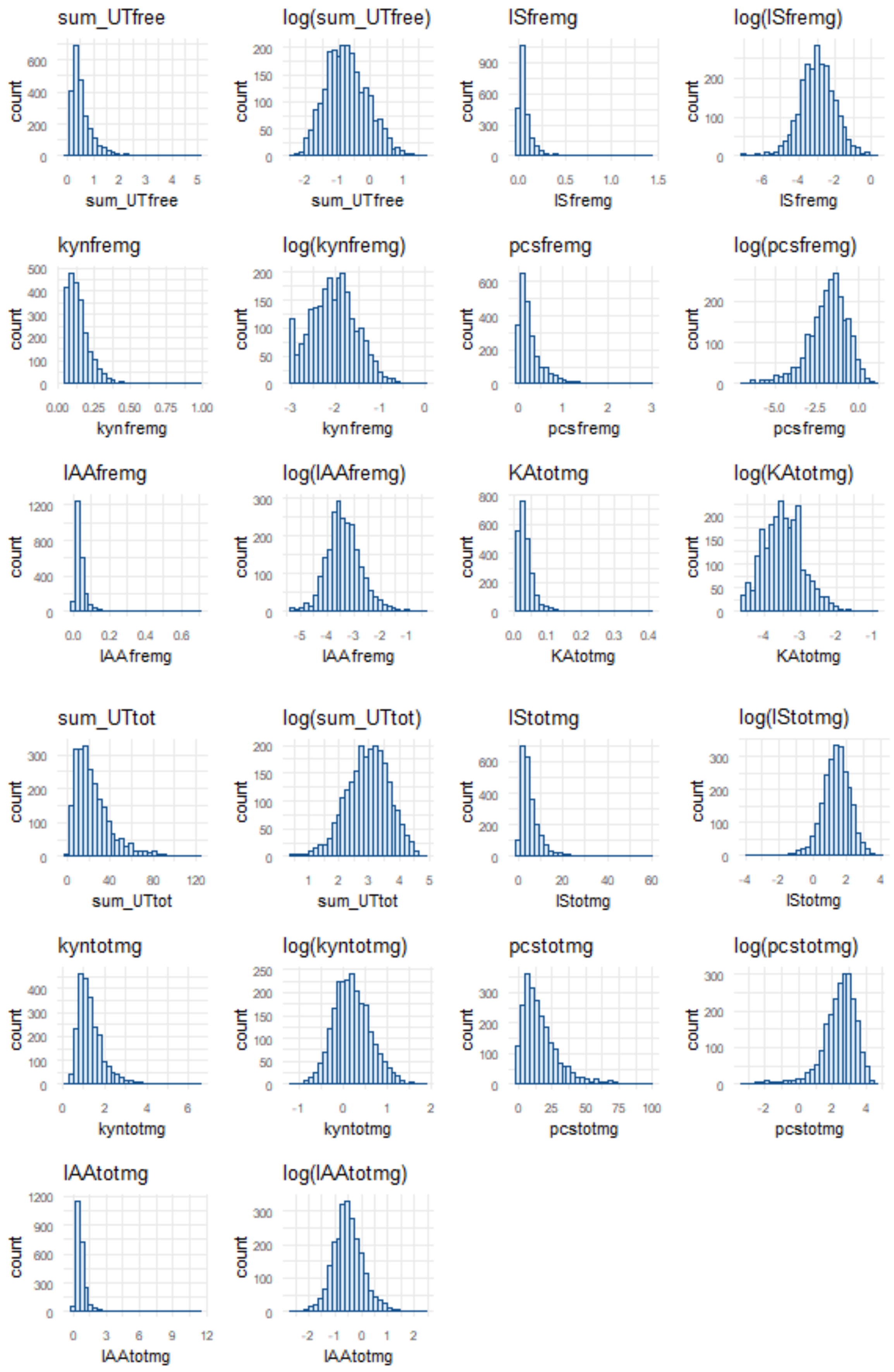
sum_UTfree
log(sum_UTfree)
ISfremg
log(ISfremg)
kynfremg
log(kynfremg)
pcsfremg
log(pcsfremg)
IAAfremg
log(IAAfremg)
KAtotmg
log(KAtotmg)
sum_UTtot
log(sum_UTtot)
IStotmg
log(IStotmg)
kyntotmg
log(kyntotmg)
pcstotmg
log(pcstotmg)
IAAtotmg
log(IAAtotmg)
count

Figure S3. Distribution of protein-bound uraemic toxin concentration before and after log transformation.

Log total UT concentration	Dose	Mean relative difference (95% CI), reference: no furosemide prescription	
		Crude	Adjusted*
$\sum UTs_{total}$[a]	10-40 mg	**25.7% (16.9% to 35.3%)**	2.2% (-4.6% to 9.4%)
	60-120 mg	**41.9% (27.9% to 57.3%)**	7.0% (-2.9% to 17.9%)
	> 120 mg	**44.2% (28.3% to 62.1%)**	6.7% (-4.8% to 19.5%)
	Any	**32.8% (25.2% to 41.1%)**	3.9% (-2.3% to 10.4%)
Indoxyl sulphate	10-40 mg	**20.0% (10.3% to 30.5%)**	-3.5% (-10.7% to 4.2%)
	60-120 mg	**30.9% (16.1% to 47.4%)**	-3.7% (-13.7% to 7.5%)
	> 120 mg	**54.8% (35.3% to 77.4%)**	10.3% (-3.0% to 25.5%)
	Any	**28.5% (20.0% to 37.7%)**	-1.9% (-8.4% to 5.1%)
Kynurenine	10-40 mg	**6.8% (2.2% to 11.5%)**	0.8% (-3.7% to 5.5%)
	60-120 mg	**15.3% (8.2% to 22.6%)**	4.7% (-1.8% to 11.7%)
	> 120 mg	**12.3% (4.6% to 20.4%)**	0.9% (-6.5% to 8.9%)
	Any	**9.9% (6.0% to 13.8%)**	1.7% (-2.4% to 6.0%)
P-cresyl sulphate	10-40 mg	**37.4% (22.9% to 53.7%)**	8.1% (-3.5% to 21.2%)
	60-120 mg	**55.7% (32.8% to 82.6%)**	13.8% (-3.1% to 33.6%)
	> 120 mg	**51.7% (26.7% to 81.7%)**	10.6% (-8.4% to 33.5%)
	Any	**44.3% (31.8% to 58.2%)**	9.7% (-0.8% to 21.4%)
Indole-3-acetic acid	10-40 mg	4.3% (-1.9% to 10.8%)	2.8% (-3.6% to 9.7%)
	60-120 mg	-1.2% (-9.4% to 7.8%)	-2.3% (-10.9% to 7.1%)
	> 120 mg	-0.3% (-9.7% to 10.0%)	0.4% (-9.9% to 11.7%)
	Any	2.0% (-2.9% to 7.3%)	1.3% (-4.4% to 7.4%)
Kynurenic acid	10-40 mg	**10.1% (4.0% to 16.6%)**	-0.2% (-5.2% to 4.9%)
	60-120 mg	**18.9% (9.5% to 29.0%)**	0.4% (-6.6% to 7.9%)
	> 120 mg	**11.3% (1.5% to 22.1%)**	**-8.2% (-15.6% to -0.1%)**
	Any	**12.4% (7.3% to 17.8%)**	-1.2% (-5.5% to 3.4%)

Figure S4. Mean relative difference in total uraemic toxin concentration, as a function of furosemide dose level category and furosemide status (reference: no furosemide prescription).

95%CIs that excluded 0% are given in bold type.
[a] The sum of total UTs, including the free fraction of indoxyl sulphate, kynurenine, p-cresyl sulphate, indole-3-acetic acid and kynurenic acid
*Adjusted for age, sex, the total number of co-prescribed medications, the number of potential OAT1/3 inhibitors, the history of AKI and CV disease, serum CRP and albumin levels, diabetes, BMI, smoking status, uACR, and eGFR.
Abbreviations: AKI, acute kidney injury; BMI, body mass index; CI, confidence interval; CRP, c-reactive protein; CV, cardiovascular; eGFR, estimated glomerular filtration rate; OAT1/3, organic anion transporters 1 and 3; uACR, urinary albumin-to-creatinine ratio; UT, uraemic toxin

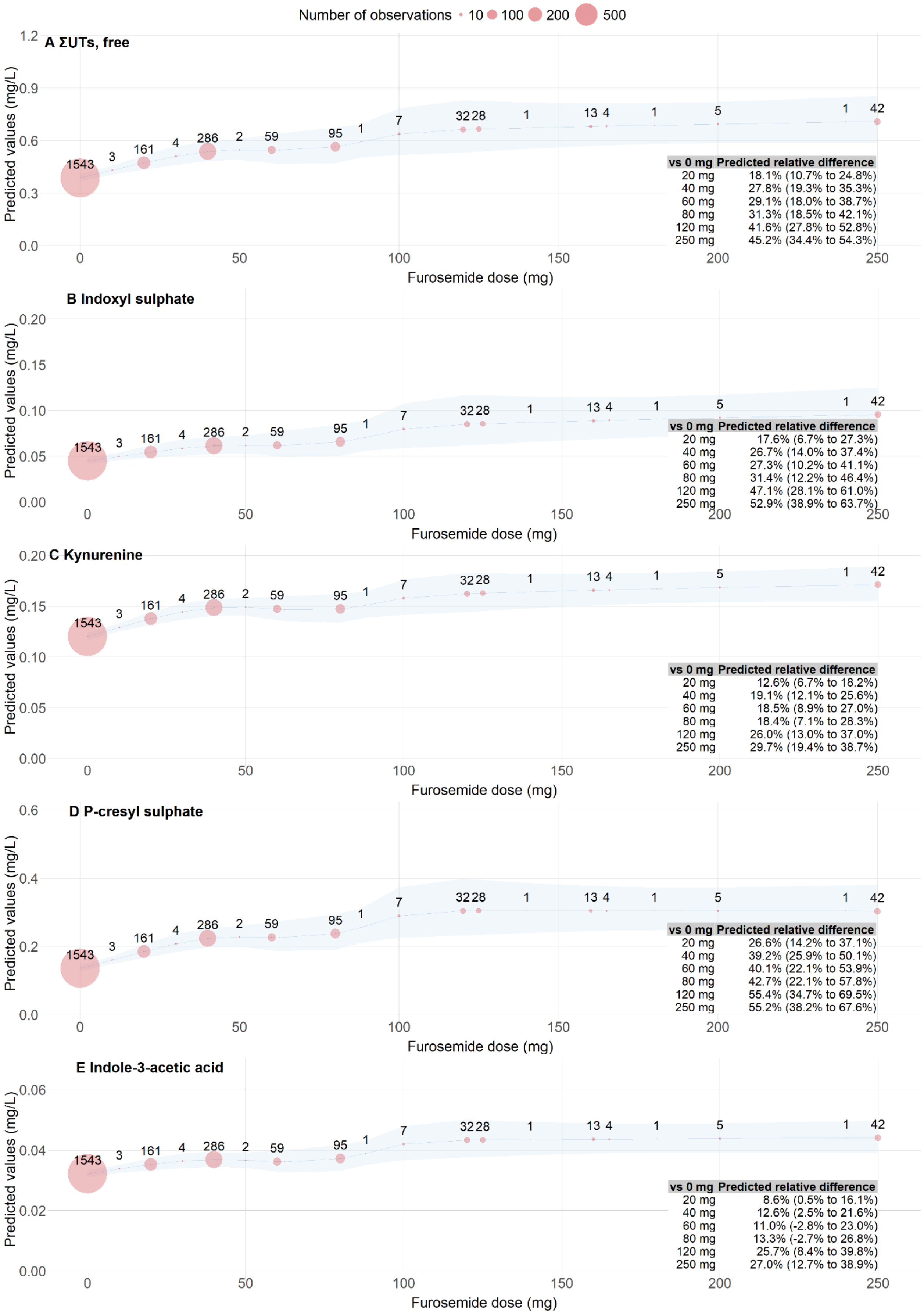

Figure S5. Predicted protein-bound uraemic toxin concentrations as a smooth function of furosemide dose level, crude model

The size of red circles in the plots are proportional to the number of observations at each data point, with the exact number of observations indicated above each circle. A total of 54 observations are not shown (distributed between the doses of 290 mg and 1000 mg).

For each plot, a table presents the predicted relative difference between no furosemide prescription and doses of 20, 40, 60, 80, 120, and 250 mg—doses prescribed to more than 30 patients.

Furosemide dose was modelled with natural splines with knots at 40mg, 80mg, 100mg and 120 mg.

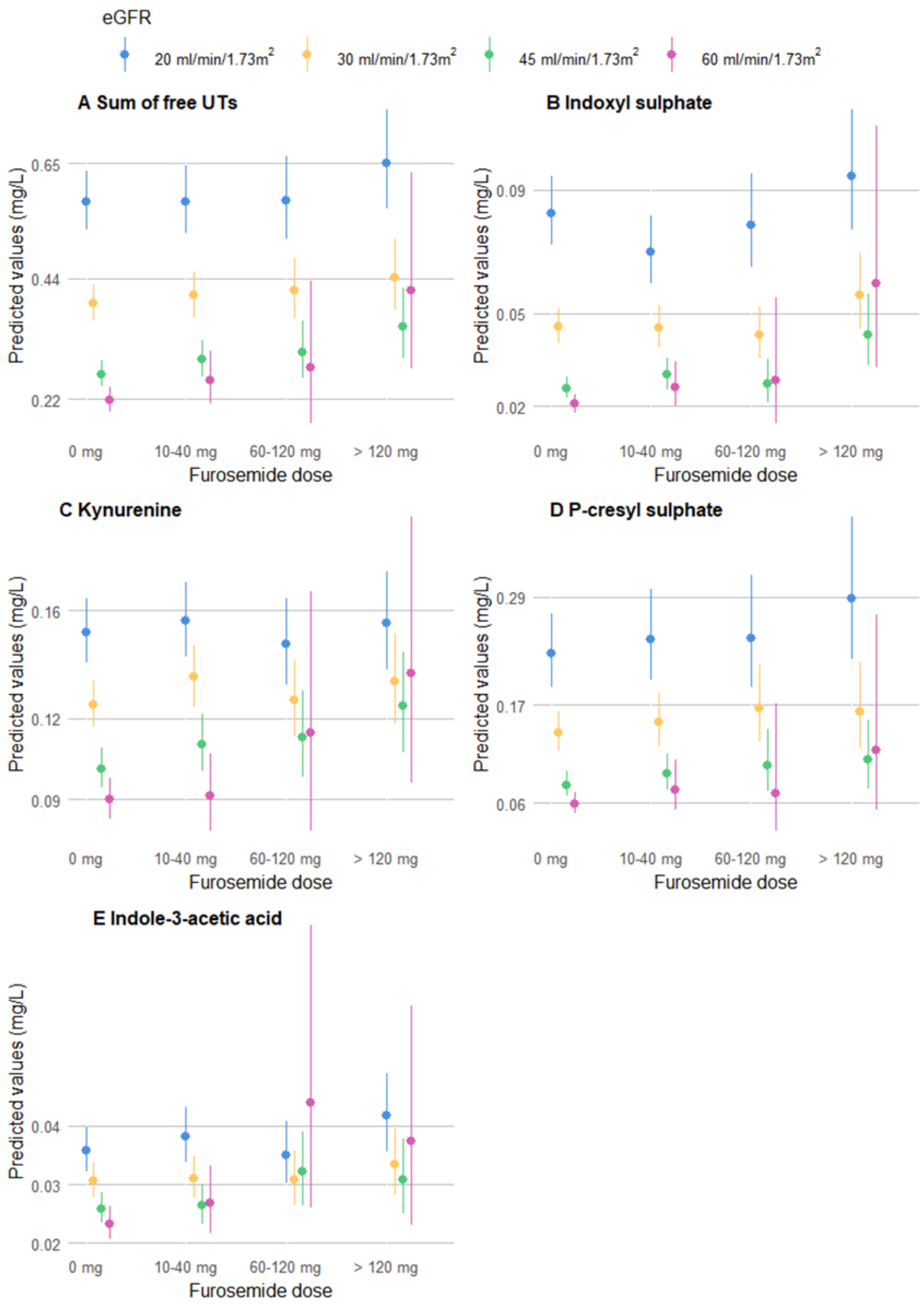

Figure S6. Predicted free uraemic toxin concentrations as a function of furosemide dose level category, stratified by different eGFR levels.

Table S4. Mean relative difference in free uraemic toxin levels, as a function of the furosemide dose level category and furosemide status and after further adjustment for uric acid.

Log free UT level	Dose	Mean relative difference (95% CI), reference: no furosemide prescription	
		Adjusted*	Adjusted*+ Uric Acid
**ΣUTs$_{free}$[a]**	**10-40 mg**	5.0% (-1.2% to 11.6%)	4.8% (-1.4% to 11.5%)
	**60-120 mg**	6.3% (-2.6% to 15.8%)	5.9% (-3.0% to 15.5%)
	**>120 mg**	**19.1% (7.7% to 31.9%)**	**18.4% (7.0% to 31.1%)**
Indoxyl sulphate	**10-40 mg**	-0.5% (-9.1% to 9.0%)	-0.9% (-9.4% to 8.5%)
	**60-120 mg**	-0.4% (-12.4% to 13.1%)	-1.1% (-13.1% to 12.4%)
	**>120 mg**	**31.9% (13.5% to 53.3%)**	**30.5% (12.2% to 51.6%)**
Kynurenine	**10-40 mg**	**6.4% (1.1% to 12.0%)**	**6.0% (0.7% to 11.6%)**
	**60-120 mg**	2.3% (-4.9% to 10.1%)	1.8% (-5.4% to 9.5%)
	**>120 mg**	**9.3% (0.4% to 19.0%)**	**8.5% (-0.4% to 18.2%)**
P-cresyl sulphate	**10-40 mg**	11.0% (-1.6% to 25.1%)	11.1% (-1.6% to 25.2%)
	**60-120 mg**	16.0% (-2.2% to 37.3%)	15.7% (-2.4% to 37.2%)
	**>120 mg**	**29.3% (6.1% to 57.8%)**	**28.9% (5.7% to 57.3%)**
Indole-3-acetic acid	**10-40 mg**	4.4% (-2.8% to 12.1%)	3.9% (-3.2% to 11.5%)
	**60-120 mg**	4.8% (-5.2% to 15.8%)	4.3% (-5.7% to 15.3%)
	**>120 mg**	**16.9% (4.0% to 31.5%)**	**16.0% (3.0% to 30.5%)**

95%CIs that excluded 0% are given in bold type.

[a] The sum of free UTs, including the free fraction of indoxyl sulphate, kynurenine, p-cresyl sulphate and indole-3-acetic acid.

*Adjusted for age, sex, the total number of co-prescribed medications, the number of potential OAT1/3 inhibitors, the history of AKI and CV disease, serum CRP and albumin levels, diabetes, BMI, smoking status, uACR, and eGFR.

Abbreviations: AKI, acute kidney injury; BMI, body mass index; CI, confidence interval; CRP, c-reactive protein; CV, cardiovascular; eGFR, estimated glomerular filtration rate; OAT1/3, organic anion transporters 1 and 3; uACR, urinary albumin-to-creatinine ratio; UT, uraemic toxin

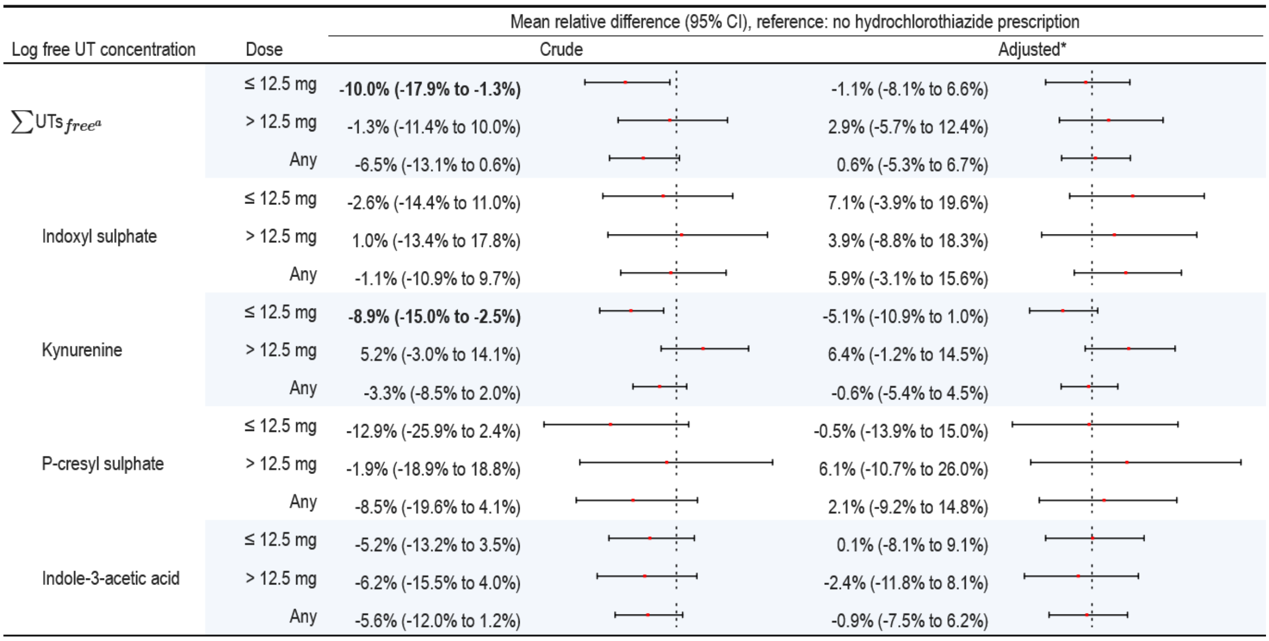

Figure S7. Mean relative difference in free uraemic toxin levels, as a function of the hydrochlorothiazide dose level category (reference: no hydrochlorothiazide prescription).

95%CIs that excluded 0% are given in bold type.

[a] The sum of free UTs, including the free fraction of indoxyl sulphate, kynurenine, p-cresyl sulphate and indole-3-acetic acid

*Adjusted for age, sex, the total number of co-prescribed medications, the number of potential OAT1/3 inhibitors, the history of AKI and CV disease, serum CRP and albumin levels, diabetes, BMI, smoking status, uACR, and eGFR.
Abbreviations: AKI, acute kidney injury; BMI, body mass index; CI, confidence interval; CRP, c-reactive protein; CV, cardiovascular; eGFR, estimated glomerular filtration rate; OAT1/3, organic anion transporters 1 and 3; uACR, urinary albumin-to-creatinine ratio; UT, uraemic toxin

		Mean relative difference (95% CI), reference: no hydrochlorothiazide prescription	
Log total UT concentration	Dose	Crude	Adjusted*
$\sum UTs_{total^a}$	≤ 12.5 mg	-6.2% (-14.9% to 3.4%)	1.0% (-7.1% to 9.7%)
	> 12.5 mg	-2.9% (-13.4% to 9.0%)	0.9% (-8.6% to 11.4%)
	Any	-4.9% (-11.9% to 2.7%)	0.9% (-5.6% to 8.0%)
Indoxyl sulphate	≤ 12.5 mg	1.7% (-9.0% to 13.7%)	7.1% (-2.6% to 17.7%)
	> 12.5 mg	-2.0% (-14.0% to 11.7%)	-2.0% (-12.3% to 9.6%)
	Any	0.2% (-8.2% to 9.4%)	3.4% (-4.2% to 11.5%)
Kynurenine	≤ 12.5 mg	-3.5% (-8.9% to 2.1%)	-3.0% (-8.2% to 2.5%)
	> 12.5 mg	1.9% (-4.8% to 9.1%)	2.4% (-4.1% to 9.4%)
	Any	-1.4% (-5.7% to 3.3%)	-0.8% (-5.2% to 3.7%)
P-cresyl sulphate	≤ 12.5 mg	-8.9% (-21.3% to 5.7%)	-1.0% (-13.7% to 13.5%)
	> 12.5 mg	-2.6% (-18.1% to 16.0%)	2.7% (-12.7% to 20.9%)
	Any	-6.3% (-16.7% to 5.3%)	0.5% (-10.1% to 12.3%)
Indole-3-acetic acid	≤ 12.5 mg	-1.5% (-9.0% to 6.6%)	-0.6% (-8.1% to 7.6%)
	> 12.5 mg	-6.3% (-14.7% to 2.8%)	-4.1% (-12.6% to 5.2%)
	Any	-3.5% (-9.4% to 2.7%)	-2.0% (-8.0% to 4.4%)
Kynurenic acid	≤ 12.5 mg	4.0% (-3.5% to 12.0%)	6.3% (0.0% to 13.0%)
	> 12.5 mg	-0.3% (-8.7% to 8.9%)	1.5% (-5.6% to 9.2%)
	Any	2.2% (-3.7% to 8.4%)	4.3% (-0.7% to 9.6%)

Figure S8. Mean relative difference in total uraemic toxin levels, as a function of the hydrochlorothiazide dose level category (reference: no hydrochlorothiazide prescription).

95%CIs that excluded 0% are given in bold type.

[a] The sum of total UTs, including the free fraction of indoxyl sulphate, kynurenine, p-cresyl sulphate, indole-3-acetic acid and kynurenic acid

*Adjusted for age, sex, the total number of concomitant prescription medications, the number of potential OAT1/3 inhibitors, the history of AKI and CV disease, serum CRP and albumin levels, diabetes, BMI, smoking status, uACR, and eGFR.

Abbreviations: AKI, acute kidney injury; BMI, body mass index; CI, confidence interval; CRP, c-reactive protein; CV, cardiovascular; eGFR, estimated glomerular filtration rate; OAT1/3, organic anion transporters 1 and 3; uACR, urinary albumin-to-creatinine ratio; UT, uraemic toxin

Supplementary Acknowledgments

Alsace : Prs T. Hannedouche et B. Moulin (CHU, Strasbourg), Dr A. Klein (CH Colmar) Aquitaine : Pr C. Combe (CHU, Bordeaux), Dr J.P. Bourdenx (Clinique St Augustin, Bordeaux), Dr A. Keller, Dr C. Delclaux (CH, Libourne), Dr B. Vendrely (Clinique St Martin, Pessac), Dr B. Deroure (Clinique Delay, Bayonne), Dr A. Lacraz (CH, Bayonne) Basse Normandie : Dr T. Lobbedez (CHU, Caen), Dr I. Landru (CH, Lisieux) Ile de France : Pr Z. Massy (CHU, Boulogne – Billancourt), Pr P. Lang (CHU, Créteil), Dr X. Belenfant (CH, Montreuil), Pr E. Thervet (CHU, Paris), Dr P. Urena (Clinique du Landy, St Ouen), Dr M. Delahousse (Hôpital Foch, Suresnes) Languedoc – Roussillon : Dr C. Vela (CH, Perpignan) Limousin : Pr M. Essig, Dr D. Clément (CHU, Limoges) Lorraine : Dr H. Sekhri, Dr M. Smati (CH, Epinal) Dr M. Jamali, Dr B. Hacq (Clinique Louis Pasteur, Essey-les-Nancy), Dr V. Panescu, Dr M. Bellou (Polyclinique de Gentilly, Nancy), Pr Luc Frimat (CHU, Vandœuvre-les-Nancy) Midi-Pyrénées : Pr N Kamar (CHU, Toulouse) Nord-Pas-de-Calais : Prs C. Noël et F. Glowacki (CHU, Lille), Dr N. Maisonneuve (CH, Valenciennes), Dr R. Azar (CH, Dunkerque), Dr M. Hoffmann (Hôpital privé La Louvière, Lille) Pays-de-la Loire : Pr M. Hourmant (CHU, Nantes), Dr A. Testa (Centre de dialyse, Rezé), Dr D. Besnier (CH, St Nazaire) Picardie : Pr G. Choukroun (CHU, Amiens), Dr G. Lambrey (CH, Beauvais) Provence-Alpes - Côte d'Azur : Pr S. Burtey (CHU, Marseille), Dr G. Lebrun (CH, Aix-en-Provence), Dr E. Magnant (Polyclinique du Parc Rambot, Aix-en-Provence) Rhône-Alpes : Pr M. Laville, Pr D. Fouque (CHU, Lyon-Sud) et L. Juillard (CHU Edouard Herriot, Lyon), Dr C. Chazot (Centre de rein artificiel Tassin Charcot, Ste Foy-les-Lyon), Pr P. Zaoui (CHU, Grenoble), Dr F. Kuentz (Centre de santé rénale, Grenoble).

List of biological resources centers:

The authors would like to thank the teams of all the biological resources centers that participated in the CKD-REIN project:

- Biobanque de Picardie, CRB du Centre Hospitalier Universitaire Amiens Picardie, 1 Rond-Point du Pr Christian Cabrol, 80054 Amiens Cedex 1 (BRIF number: BB-0033-00017)
- NeuroBioTec, CRB des Hospices Civils de Lyon Groupement Hospitalier Est, Hôpital Neurologique, 59 Boulevard Pinel, 69677 Bron Cedex (BRIF number: BB-0033-00046)
- Centre de ressources biologiques du Centre Hospitalier Universitaire de Nantes Hôtel Dieu, Institut de biologie, 9, quai Moncousu, 44093 Nantes Cedex 1 (BRIF number: BB-0033-00040)
- Centre de ressources biologiques du Centre Hospitalier Universitaire Grenoble Alpes, Boulevard de la Chantourne, CS 10217, 38700 La Tronche (BRIF number: BB-0033-00069)
- Centre de ressources biologiques du Centre Hospitalier Régional Universitaire de Nancy, Hôpitaux de Brabois, Bâtiment Recherche Rue du Morvan, 54500 Vandoeuvre-les-Nancy (BRIF number: BB-0033-00035)

- Service de Néphrologie, Centre Hospitalier de Perpignan, 20 Avenue du Languedoc, 66046 Perpignan Cedex 9
- Plateforme de Ressources Biologiques, Hôpital Henri Mondor, 51 avenue du Maréchal de Lattre de Tassigny, 94000 Créteil (BRIF number: BB-0033-00021)
- CIC-1435, Centre d'Investigation Clinique Plurithématique, Centre Hospitalier Universitaire de Limoges, 2 Avenue Martin Luther King, 87042 Limoges Cedex
- Plateforme de Ressources Biologiques de l'Hôpital européen Georges-Pompidou, 20-40 rue Leblanc, 75015 Paris (BRIF number: BB-0033-00063)
- Etablissement Français du sang Hauts de France – Normandie, Site de Bois-Guillaume 609, chemin de la Bretèque, 76235 Bois-Guillaume
- Etablissement Français du sang Nouvelle Aquitaine, site Pellegrin, Place Amélie Raba Léon, CS 21010, 33075 Bordeaux Cedex
- Etablissement Français du sang Hauts de France – Normandie, Site de Loos-Eurasanté, Avenue Pierre Mauroy, Parc Eurasante Epi-de Soil, 59120 Loos
- Etablissement Français du sang Ile de France, Site Avicenne, Hopital Avicenne porte 8, 125 route de Stalingrad, 93009 Bobigny
- Etablissement Français du sang Occitanie, Site de Toulouse, 75 rue de Lisieux, 31300 Toulouse
- Etablissement Français du sang Grand-Est, Site de Colmar, 6 rue du Hohnack, 68025 Colmar Cedex
- Etablissement Français du sang Grand-Est, Site de Metz, 6 rue des Dames de Metz, 57000 Metz
- Etablissement Français du sang PACA-Corse, Site de Marseille, 149, boulevard Baille, 13392 Marseille Cedex 05

STROBE Checklist

STROBE Statement—Checklist of items that should be included in reports of ***cross-sectional studies***

	Item No	Recommendation	Page No.
**Title and abstract**	1	(*a*) Indicate the study's design with a commonly used term in the title or the abstract	1
		(*b*) Provide in the abstract an informative and balanced summary of what was done and what was found	3
**Introduction**			
Background/rationale	2	Explain the scientific background and rationale for the investigation being reported	5-6
Objectives	3	State specific objectives, including any prespecified hypotheses	6
**Methods**			
Study design	4	Present key elements of study design early in the paper	7
Setting	5	Describe the setting, locations, and relevant dates, including periods of recruitment, exposure, follow-up, and data collection	7
Participants	6	(*a*) Give the eligibility criteria, and the sources and methods of selection of participants	7
Variables	7	Clearly define all outcomes, exposures, predictors, potential confounders, and effect modifiers. Give diagnostic criteria, if applicable	8-9
Data sources/ measurement	8*	For each variable of interest, give sources of data and details of methods of assessment (measurement). Describe comparability of assessment methods if there is more than one group	8-9
Bias	9	Describe any efforts to address potential sources of bias	10
Study size	10	Explain how the study size was arrived at	7
Quantitative variables	11	Explain how quantitative variables were handled in the analyses. If applicable, describe which groupings were chosen and why	9
Statistical methods	12	(*a*) Describe all statistical methods, including those used to control for confounding	9
		(*b*) Describe any methods used to examine subgroups and interactions	11
		(*c*) Explain how missing data were addressed	10
		(*d*) If applicable, describe analytical methods taking account of sampling strategy	
		(*e*) Describe any sensitivity analyses	11
**Results**			

Participants	13*	(a) Report numbers of individuals at each stage of study—eg numbers potentially eligible, examined for eligibility, confirmed eligible, included in the study, completing follow-up, and analysed	11
		(b) Give reasons for non-participation at each stage	11
		(c) Consider use of a flow diagram	11
Descriptive data	14*	(a) Give characteristics of study participants (eg demographic, clinical, social) and information on exposures and potential confounders	11
		(b) Indicate number of participants with missing data for each variable of interest	11 (Table 1)
Outcome data	15*	Report numbers of outcome events or summary measures	11-12
Main results	16	(*a*) Give unadjusted estimates and, if applicable, confounder-adjusted estimates and their precision (eg, 95% confidence interval). Make clear which confounders were adjusted for and why they were included	12
		(*b*) Report category boundaries when continuous variables were categorized	12
		(*c*) If relevant, consider translating estimates of relative risk into absolute risk for a meaningful time period	
Other analyses	17	Report other analyses done—eg analyses of subgroups and interactions, and sensitivity analyses	13
**Discussion**			
Key results	18	Summarise key results with reference to study objectives	14
Limitations	19	Discuss limitations of the study, taking into account sources of potential bias or imprecision. Discuss both direction and magnitude of any potential bias	18
Interpretation	20	Give a cautious overall interpretation of results considering objectives, limitations, multiplicity of analyses, results from similar studies, and other relevant evidence	14-18
Generalisability	21	Discuss the generalisability (external validity) of the study results	14-18
**Other information**			
Funding	22	Give the source of funding and the role of the funders for the present study and, if applicable, for the original study on which the present article is based	19

Supplementary References

S1. Deguchi T, Ohtsuki S, Otagiri M, et al. Major role of organic anion transporter 3 in the transport of indoxyl sulfate in the kidney. *Kidney International*. 2002;61(5):1760-1768. doi:10.1046/j.1523-1755.2002.00318.x

S2. Deguchi T, Kusuhara H, Takadate A, Endou H, Otagiri M, Sugiyama Y. Characterization of uremic toxin transport by organic anion transporters in the kidney. *Kidney International*. 2004;65(1):162-174. doi:10.1111/j.1523-1755.2004.00354.x

S3. Tahara H, Shono M, Kusuhara H, et al. Molecular Cloning and Functional Analyses of OAT1 and OAT3 from Cynomolgus Monkey Kidney. *Pharm Res*. 2005;22(4):647-660. doi:10.1007/s11095-005-2503-0

S4. Jansen J, Fedecostante M, Wilmer MJ, et al. Bioengineered kidney tubules efficiently excrete uremic toxins. *Sci Rep*. 2016;6:26715. doi:10.1038/srep26715

S5. Kong F, Pang X, Zhong K, et al. Increased Plasma Exposures of Conjugated Metabolites of Morinidazole in Renal Failure Patients: A Critical Role of Uremic Toxins. *Drug Metab Dispos*. 2017;45(6):593-603. doi:10.1124/dmd.116.074492

S6. Enomoto A, Takeda M, Taki K, et al. Interactions of human organic anion as well as cation transporters with indoxyl sulfate. *European Journal of Pharmacology*. 2003;466(1):13-20. doi:10.1016/S0014-2999(03)01530-9

S7. Enomoto A, Takeda M, Tojo A, et al. Role of Organic Anion Transporters in the Tubular Transport of Indoxyl Sulfate and the Induction of its Nephrotoxicity. *Journal of the American Society of Nephrology*. 2002;13(7):1711. doi:10.1097/01.ASN.0000022017.96399.B2

S8. Murine renal organic anion transporters mOAT1 and mOAT3 facilitate the transport of neuroactive tryptophan metabolites. doi:10.1152/ajpcell.00619.2004

S9. Uwai Y, Honjo H, Iwamoto K. Interaction and transport of kynurenic acid via human organic anion transporters hOAT1 and hOAT3. *Pharmacological Research*. 2012;65(2):254-260. doi:10.1016/j.phrs.2011.11.003

S10. Tust M, Müller JP, Fischer D, Gründemann D. SLC22A11 Inserts the Uremic Toxins Indoxyl Sulfate and P-Cresol Sulfate into the Plasma Membrane. *Int J Mol Sci*. 2023;24(20):15187. doi:10.3390/ijms242015187

S11. Tsutsumi Y, Deguchi T, Takano M, Takadate A, Lindup WE, Otagiri M. Renal Disposition of a Furan Dicarboxylic Acid and Other Uremic Toxins in the Rat. *J Pharmacol Exp Ther*. 2002;303(2):880-887. doi:10.1124/jpet.303.2.880

S12. Tang J, Shen H, Zhao X, et al. Endogenous Plasma Kynurenic Acid in Human: A Newly Discovered Biomarker for Drug-Drug Interactions Involving Organic Anion Transporter 1 and 3 Inhibition. *Drug Metab Dispos*. 2021;49(12):1063-1069. doi:10.1124/dmd.121.000486

S13. Granados JC, Ermakov V, Maity K, Vera DR, Chang G, Nigam SK. The kidney drug transporter OAT1 regulates gut microbiome–dependent host metabolism. *JCI Insight*. 8(2):e160437. doi:10.1172/jci.insight.160437

S14. Fujita T, Ishihara K, Yasuda S, et al. In vivo kinetics of indoxyl sulfate in humans and its renal interaction with angiotensin-converting enzyme inhibitor quinapril in rats. *J Pharmacol Exp Ther*. 2012;341(3):626-633. doi:10.1124/jpet.111.187732

S15. Taniguchi T, Omura K, Motoki K, et al. Hypouricemic agents reduce indoxyl sulfate excretion by inhibiting the renal transporters OAT1/3 and ABCG2. *Sci Rep*. 2021;11:7232. doi:10.1038/s41598-021-86662-9

S16. Lai Q, Zhu X, Zhang L, et al. Inhibition of OAT1/3 and CMPF uptake attenuates myocardial ischemia-induced chronic heart failure via decreasing fatty acid oxidation and the therapeutic effects of ruscogenin. *Translational Research*. 2023;261:1-15. doi:10.1016/j.trsl.2023.06.001

S17. Latkovskis G, Makarova E, Mazule M, et al. Loop diuretics decrease the renal elimination rate and increase the plasma levels of trimethylamine-N-oxide. *Br J Clin Pharmacol*. 2018;84(11):2634-2644. doi:10.1111/bcp.13728
